# Effect of the presence of an aquarium in the waiting area on the stress, anxiety and mood of adult dental patients: A controlled clinical trial

**DOI:** 10.1371/journal.pone.0258118

**Published:** 2021-10-12

**Authors:** Andrea Lundberg, Murali Srinivasan

**Affiliations:** Clinic of General, Special Care, and Geriatric Dentistry, Centre of Dental Medicine, University of Zurich, Zurich, Switzerland; Iowa State University, UNITED STATES

## Abstract

**Background:**

Interaction with fish is known to reduce stress and anxiety in humans.

**Objective:**

This trial evaluated the effect of an aquarium present in a geriatric dental clinic waiting-area (WA) on blood pressure (BP), heart-rate (HR), anxiety, and mood of waiting patients.

**Methods:**

Participants were recruited into three groups: control (CG): WA without aquarium; partially-stocked aquarium (PSA): aquarium without fish; fully-stocked aquarium (FSA): aquarium with fish. BP and HR of the participants were recorded upon arrival and after 20-minutes of waiting, along with anxiety [State trait anxiety inventory (STAI-6)] and mood [Feeling scale (FS), Felt arousal scale (FAS)] scores. A purpose-built questionnaire evaluated the subjective assessment of the participants’ experience in the WA. ANOVA with repeated measures and nonparametric tests were used for statistical analysis (p<0.05).

**Results:**

392 patients (mean age: 65.07±16.9y) completed this trial. There was an effect of time on the BP [systolic: F(1, 120) = 44.82, p<0.001; diastolic: F(1, 120) = 25.10, p<0.001] and HR [F(1, 120) = 40.94, p<0.001]. No effect of groups on BP [systolic: F(1, 120) = 1.01, p = 0.32; diastolic: F(1, 120) = 0.01, p = 0.92] was revealed, but a decrease of HR [F(1, 120) = 21.59, p<0.001]. No effect of time*group on BP [systolic: F(1, 120) = 0.89, p = 0.35; diastolic: F(1, 120) = 0.31, p = 0.58], or HR [F(1, 120) = 1.04, p-0.31]. WA groups had no effects on the participants’ anxiety [H(2) = 2.76, p = 0.25], or mood [FS: H(2) = 2.28, p = 0.32; FAS: H(2) = 1.54, p = 0.46]. Patients rated FSA higher than others [H(2) = 20.98, p<0.001).

**Conclusions:**

There was no influence of the presence of an aquarium on the patients’ blood pressure, heart rate, anxiety, or mood.

## Introduction

Beneficial effects of interaction with animals on the reduction of stress have been reported in literature [1, 2]. It has been demonstrated that the presence of a therapy dog on a patient’s lap reduced treatment anxiety and stress, during a dental procedure [1]. Furthermore, the impact of the live fish has a positive effect on the psychological wellbeing and stress levels. After a period of observing live fish, increased relaxation, reduced anxiety and better mood was reported by participants [3]. Even by observing videotapes of fish, an increased relaxation in study participants were recorded when compared to control groups who observed blank screens or videos of humans [4]. Presence of aquariums generally tend to lower both systolic and diastolic blood pressures [3, 5, 6]. Interestingly, aquariums not only reduced stress, but also have been documented to increase the pain thresholds in participants [7]. Cognitive decline, physical disability, multimorbid status, care-resistant behaviour, general depression may all contribute to an elderly individual’s stress. This may impact the elders’ cooperativeness for medical and dental care. Aquariums in dementia units have demonstrated an improvement in the challenging behaviour, cooperativeness, and sleep of the residents; as well as an increase in the staff satisfaction [8]. Residents in dementia units sign also increased their weights significantly when an aquarium was introduced in their dining areas [9]. Furthermore similar effects of reduction in blood pressure and anxiety have been observed in non-institutionalized older adults [10]. A recent systematic review concluded that although positive effects in terms of psychological and physiological wellbeing in humans have been evidenced when interacting with fish or aquariums, scientific evidence is still scarce and further research through well-designed studies with robust methodologies were deemed necessary [11].

The objective of this controlled clinical trial was to assess the effect of an aquarium present in the waiting area on the anxiety (quantitative and subjective), and mood of the participants waiting in a university-setting dental clinic specialised for special care and geriatric dentistry. Therefore, the null hypothesis set for this controlled clinical trial was that there will be no effect of an aquarium present in the waiting area on the anxiety and mood of the participants.

## Materials and methods

The trial protocol was reviewed by the relevant ethics committee in Zurich [Kantonale Ethikkommission Zurich (KEK Zurich)], Switzerland and was decisioned that the project did not fall within the scope of the Human Research Act (HRA) and therefore did not require a formal ethics approval of the cantonal ethics committee (Basec Nr. 2020–01315). The study protocol has been registered with the ClinicalTrials.gov (ClinicalTrials.gov. ID: NCT04630600). This clinical trial has been reported according to the CONSORT (Consolidated Standards of Reporting Trials) guidelines [12].

### Trial design and participants

This study was designed as a single-blinded, monocentric, non-randomized controlled trial with an allocation ratio of 1: 1: 1. Participants were recruited if they were:

- adults ≥18 years
- able to give informed consent as documented by signature.

Participants were excluded if they were:

- already enrolled in another clinical trial.
- not willing to sign an informed consent.

### Settings and locations

This controlled clinical trial was conducted in the Clinic of General, Special care and Geriatric Dentistry in the Centre of Dental Medicine at the University of Zurich, Zurich, Switzerland. The participants were recruited from the existing patient pool of the clinic.

### Interventions

**Fig 1:**
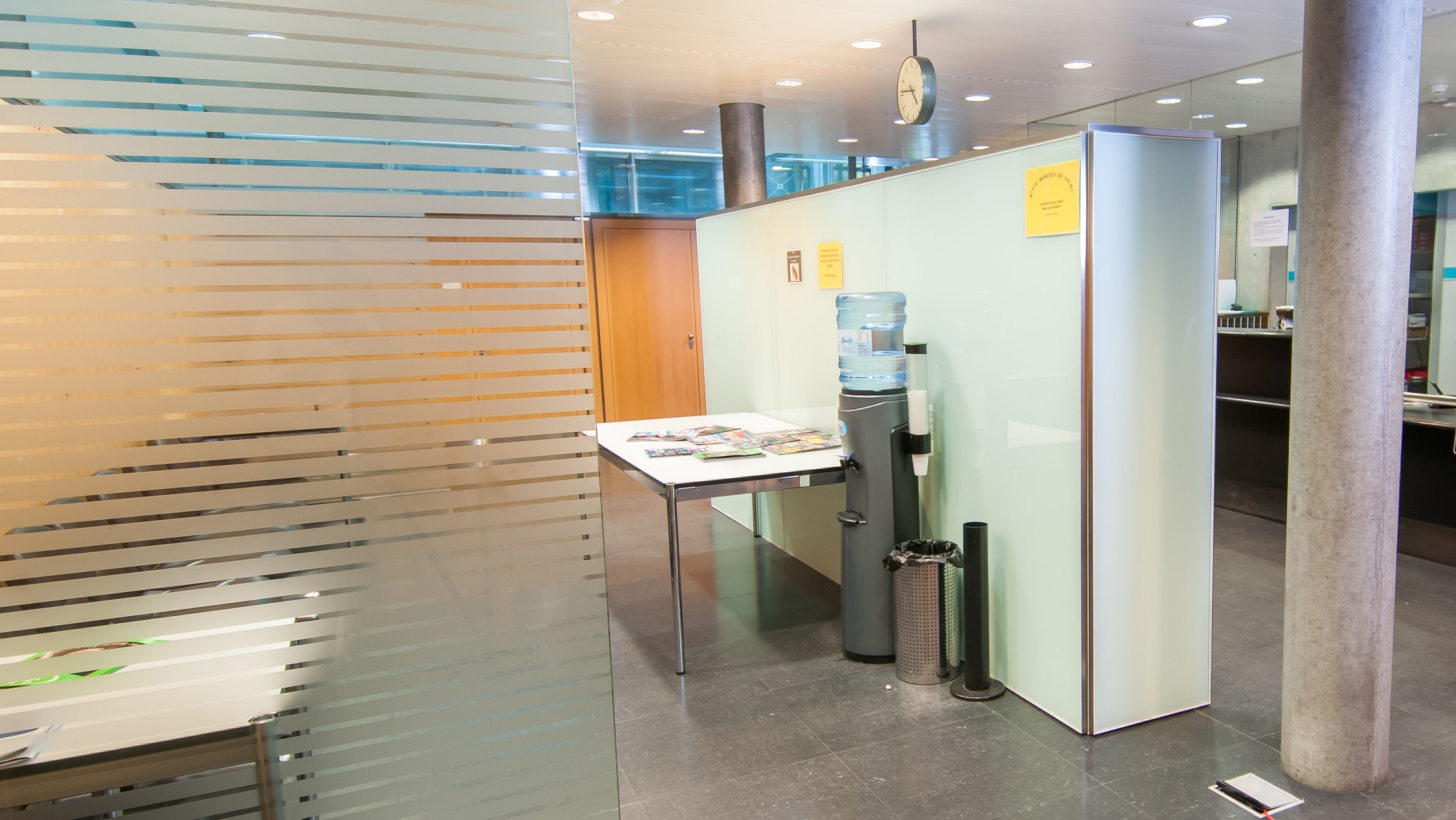
Control group waiting area without an aquarium.

**Fig 2:**
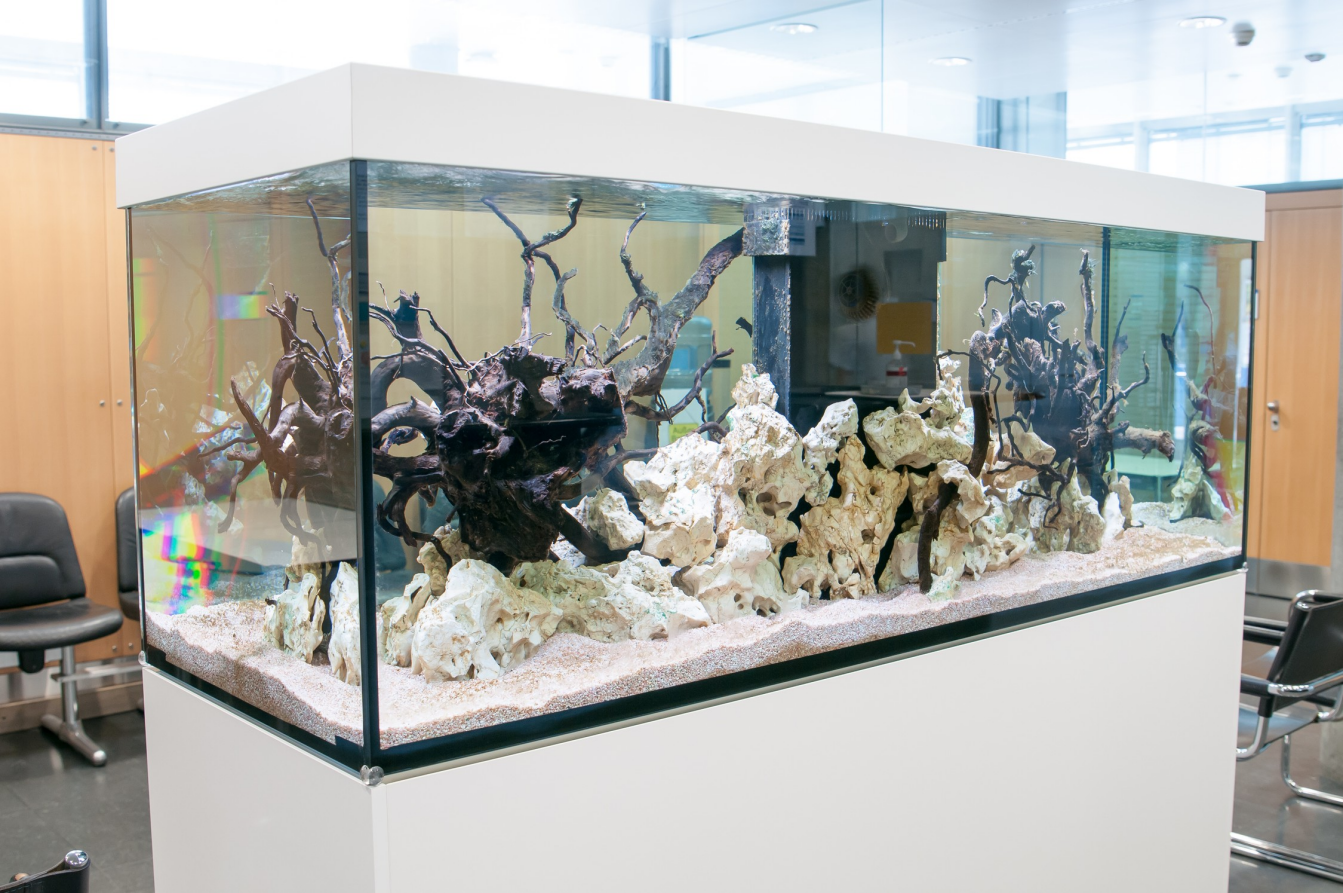
Partially-stocked aquarium without fish.

**Fig 3:**
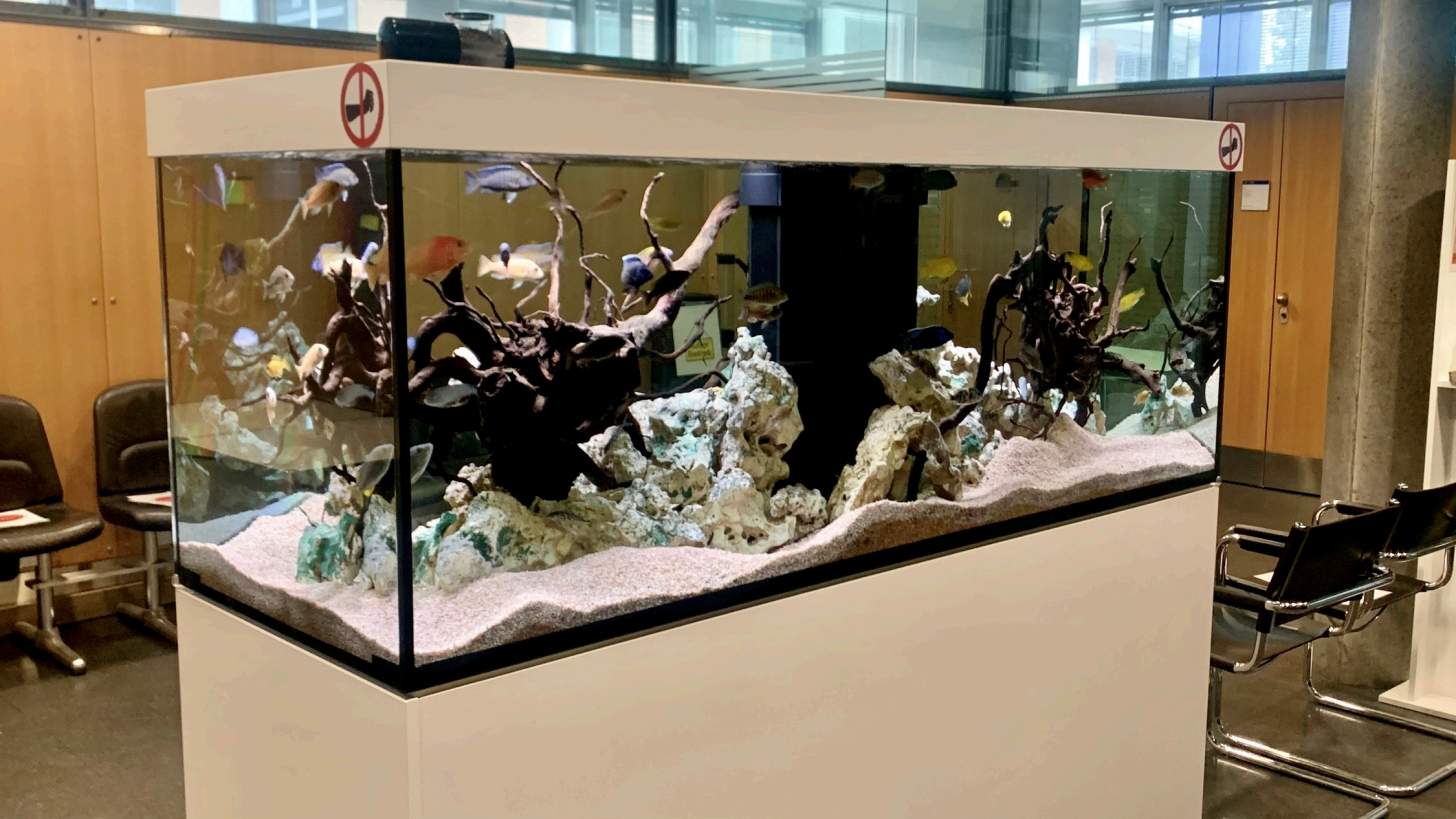
Fully-stocked aquarium with fish.

**Fig 4:**
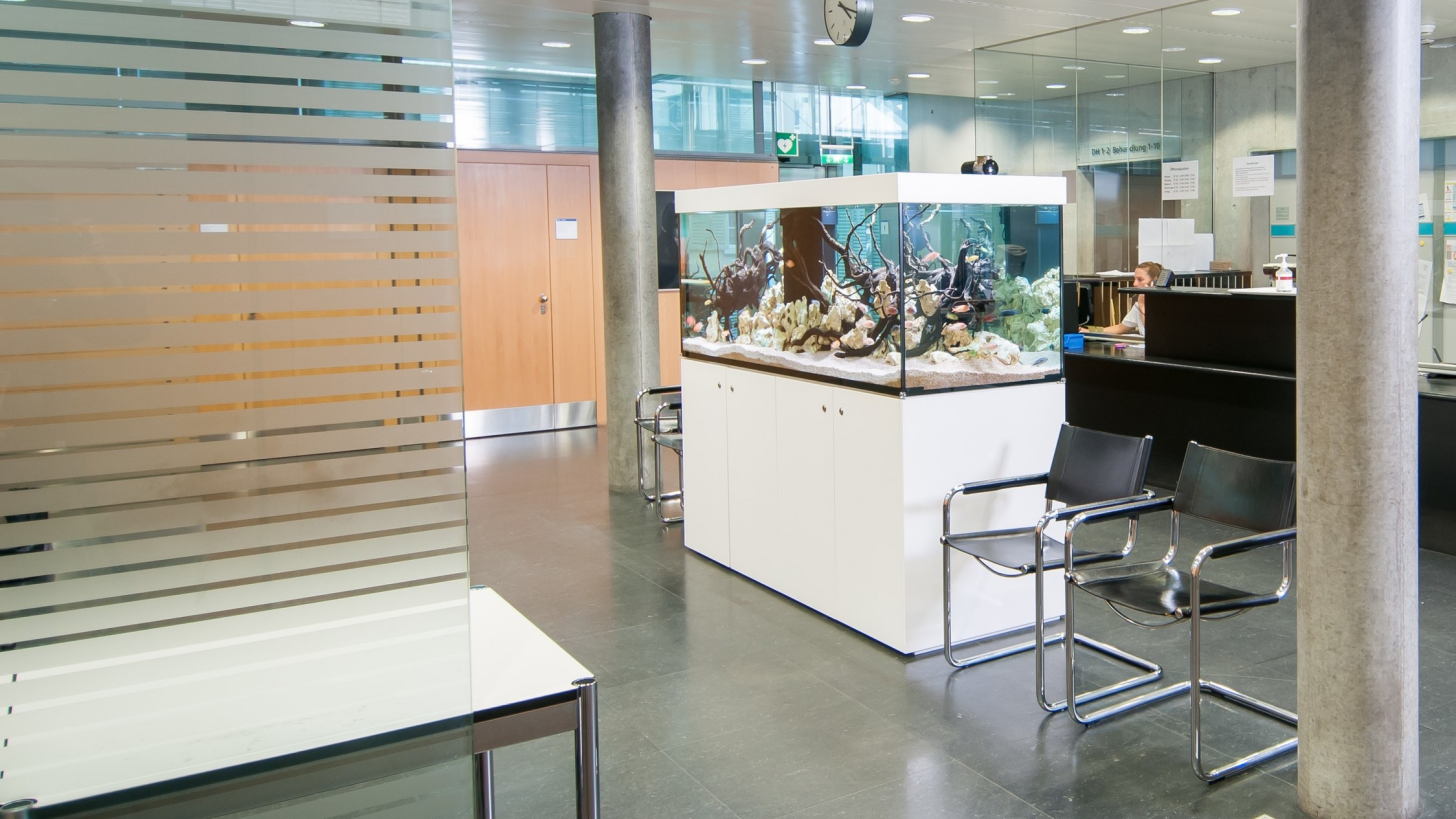
Waiting area with fully stocked aquarium with fish.

The interventions in this clinical study were the different waiting area ambiences to which the participants were subjected to during their waiting time at the clinic. The participants were sequentially recruited into one of the three intervention groups. The study groups were as follows:

- Control group (CG): The participants recruited in this group were subjected to waiting/working in the waiting area without an aquarium present (Fig 1).
- Partially-stocked aquarium group: The participants recruited in this group were subjected to waiting/working in the waiting area but with a partially-stocked aquarium present. There were no fish present in the aquarium but it was equipped with water and other decorative components (Fig 2).
- Fully-stocked aquarium group: The participants recruited in this group were subjected to waiting/working in the waiting area with a fully-stocked aquarium including fish (*sp*. *Malawi Cichlids*) (Figs 3 and 4).

### Primary outcomes/endpoints

The primary endpoints assessed in this study were the Blood pressure, heart rate, anxiety, and mood of the participants.

#### Blood Pressure (BP) and Heart Rate (HR)

The HR and BP of the participants (patients) were measured at two timepoints: T0 –upon their arrival into the dental clinic waiting area, and T1 –after 20 minutes of waiting in the waiting area. Three measurements were recorded for each timepoint and the mean of the three measurements was calculated and used for the analyses.

#### Anxiety

Anxiety was measured using a six-item State-Trait Anxiety Inventory (STAI-6) questionnaire [13]. The STAI-6 questions comprised of six questions with a 4-point Likert-scale response type (1- not at all, 2- somewhat, 3- moderately, 4- very much). The questionnaire was completed by the participants while waiting for 20 minutes and before they went to their respective planned dental treatments. The same STAI-6 questionnaire was also given to the treating clinician (therapist) to be completed in order to assess the anxiety of the patient during the treatment.

#### Mood (valence and arousal)

The mood of the participants was assessed for valence and arousal, using the feeling scale (FS) and the felt arousal scale (FAS) questionnaires, respectively [14, 15]. FS measured the valence with a scale between -5 (very bad) and +5 (very good). FAS had a scale between 1 (low arousal) and 6 (high arousal) and measured the arousal. All the questionnaires were translated in to the local language (German) as prescribed by the Medical Outcomes Trust criteria [16]. The STAI-6, FA & FAS questionnaires were translated from English to German and then back-translated to English. The translated and back-translated questionnaires were reviewed by the investigators and diagnosed for any problems in comprehension; if any problems existed, they were corrected.

### Secondary outcomes/endpoints

#### Participant’s subjective assessment of their Waiting Area Experience (WAE)

A purpose-built questionnaire comprising of five questions with a 5-point Likert-scale response type (1- strongly disagree, 2-somewhat disagree, 3- neutral, 4- somewhat agree, and 5- strongly agree) was given to the participants to be completed while waiting for 20 minutes and before they went to their respective planned dental treatments (Table 1).

**Table 1:** Questionnaire for subjectively assessing the waiting area experience of the participants.

Evaluation Statements	Strongly Disagree	Somewhat Disagree	Neutral	Somewhat Agree	Strongly Agree
1. I was very bored sitting in this waiting area.	5	4	3	2	1
2. I was stressed while sitting in this waiting area.	5	4	3	2	1
3. I enjoyed sitting in this waiting area.	1	2	3	4	5
4. The ambience of the waiting area was calming.	1	2	3	4	5
5. I would be happy to sit and wait for long durations in this waiting area.	1	2	3	4	5

### Sample size

The sample size was calculated using a freeware program (G*power 3.1.9.6 for Mac OS X) [17], from the significant results (mean and SD) reported in a previously published study with similar outcomes [3]. For BP and HR, the total sample sizes calculated were 126, and 57, with effect sizes of 0.32, and 0.49, respectively, with α = 0.05 for a power of 0.95 (1-β error probability) and assuming a normal distribution. For valence and arousal, the required total sample sizes were calculated as 41, and 30, with effect sizes of 0.58, and 0.32, respectively, with α = 0.05 for a power of 0.95 (1-β error probability) and assuming a normal distribution. Therefore, based on the calculated sample sizes and assuming a high dropout rate, a minimum sample size of 120 was fixed and a post hoc power analysis was performed for parameters with nonsignificant results to rule out type II statistical errors [18].

### Randomization and allocation

This was not a randomized trial. Participants were sequentially recruited and once the sample number was achieved for the recruiting group, the participants were then included into the subsequent study groups. The first set of participants were enrolled into CG, then into the partially-stocked aquarium group, and finally into the fully-stocked aquarium group. Although the study was not randomized, it was single-blinded as the participants were blinded to the exact intervention and the outcome. The detailed recruitment process and procedure is explained below in the study protocol and procedures section.

### Study protocol and procedures

All potential participants visiting the clinic for dental treatment were invited to participate. The details of the study were explained to the potential participant and sufficient time was given to decide. Some participants decided to participate instantly while others took longer to decide to participate. A specific time was not set for receiving consent. Upon receiving the signed informed consent from the participant, they were recruited into the study. The first set of participants were sequentially enrolled into the control group (CG). This was the group of participants who were exposed to an unmodified existing waiting area, without an aquarium. When the required number of participants had been recruited into the CG, the aquarium was installed in the middle of the waiting area. This aquarium was partially equipped, i.e., it comprised of all the components of an aquarium except the fish. As soon as the partially-stocked aquarium was ready, the enrolment of the participants for the second study group began. On recruitment of the last required participant for the partially-stocked group, the fish were brought into the aquarium. Then the final group of participants were recruited and enrolled into the third study group (fully-stocked aquarium group). For each patient enrolled in the study, the demographic information, blood pressure (BP), and heart rate (HR) were measured. BP and HR were recorded with a standard digital bedside sphygmomanometer (PMV- 2701, Nihon Kohden, Japan), three times. Later on, the study questionnaires (STAI-6, FS, FAS, and the waiting area questions) were administered to the participants. The participants were filling out the questionnaires while waiting. The study investigator (AL) was present to clarify any doubts, concerning the questionnaires, which may have risen during completion by the participants. After completion of the questionnaires, that were filled out during the 20 minutes of waiting in the waiting area, the blood pressures and heart rates were measured again, three times [19]. The patients then proceeded to their respective planned dental treatments. The dental professionals, who provided the dental care to the patients, completed the STAI-6 questionnaires based on their perceptions of the patients’ anxiety.

### Statistical methods

Mean, median, standard deviations were calculated for BP, HR, STAI-6, FS, FAS, and the waiting area questionnaires. For non-significant findings, post hoc power analyses were performed to rule out type II errors. The data was verified for normal distribution using K-S test. Data pertaining to mean systolic BP (KS test: p = 0.169), mean diastolic BP (KS test: p = 0.200), heart rate (KS test: p = 0.200) were normally distributed. ANOVA with repeated measures was performed for evaluating the intra-and intergroup differences for BP and HR with assessing interactions by time, group, and influence of group and time (group*time) with α = 0.05. Data for STAI-6 (KS-test: p<0.001), FS (KS-test: p<0.001), and FAS (KS-test: p<0.001) and the participants’ WAE (KS-test: p = 0.045) scores were not normally distributed. These were analysed using the non-parametric tests (Kruskal-Wallis H mean rank test and Chi-square median tests) with the significance set at α = 0.05. All statistical analyses were performed using a statistical software package (IBM^®^ SPSS^®^ Statistics, version 25, IBM^®^ Corporation, NY, USA).

## Results

**Fig 5:**
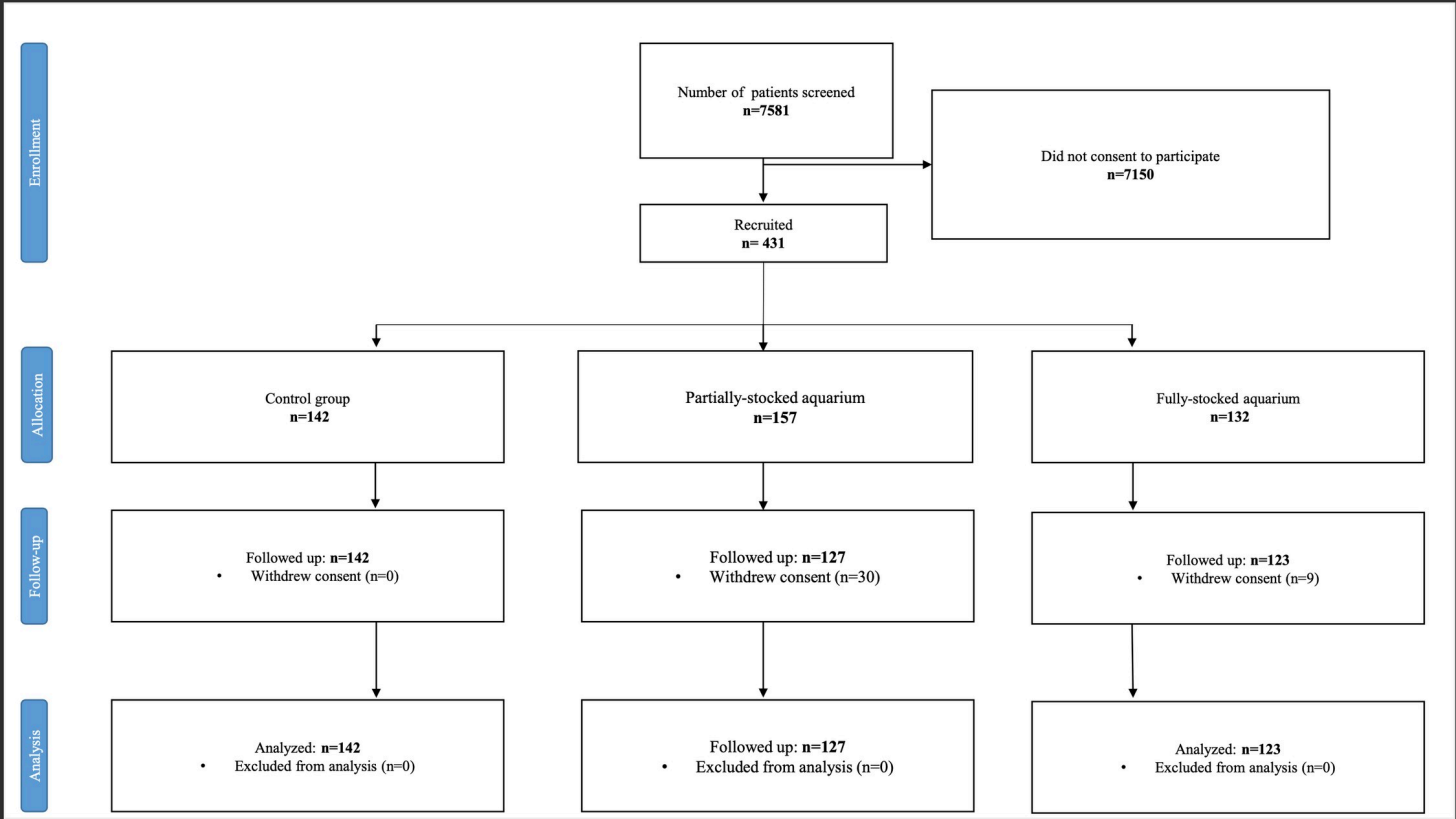
CONSORT flow diagram showing the patients’ enrolment, allocation, follow-up and analysis process. n- number; Control group- waiting area without aquarium; Intervention group #1- Waiting area with a partially-equipped aquarium; Intervention group #3- Waiting area with a fully-equipped aquarium.

A total of 7581 prospective participants from the patient population were screened, out of which 431 patients (control group: n = 142; partially-stocked group: n = 157; fully-stocked group: n = 132) consented to participate in this trial. The number of participants screened, recruited, allocated to interventions, excluded (with reasons for exclusion) and numbers analysed (n = 392), are presented in Fig 5. The participant recruitment and the study ended on 19 November 2020 after the last required participant was enrolled. The baseline characteristics of the participants are shown in Table 2.

**Table 2:** Baseline demographics of the participants.

	Total participants	Control	Partially-stocked aquarium	Fully-stocked aquarium	p-value
Number (n)	392	142	127	123	-
Sex [male {n(%)}; female {n(%)}]	210(53.6); 182(46.4)	87(61.3); 55(38.7)	60(47.2); 67(52.8)	63(51.2); 60(48.8)	0.003§
Institutionalized [n(%)]	98(25)	34(23.9)	32(25.2)	32(26.0)	0.925§
Physical disability present [n(%)]	99(25.3)	36(25.4)	34(26.8)	29(23.6)	0.844§
Number of patients dependent for care [n(%)]	59(15.1)	16(11.3)	18(14.2)	25(20.3)	0.114§
Number of patients with requiring a companion to access care [n(%)]	83(21.2)	30(21.1)	28(22.0)	25(20.3)	0.946§
Hypertensive [n(%)]	136(34.7)	44(31.0)	50(39.4)	42(34.1)	0.349§
Controlled Hypertensive [n(%)]	125(31.9)	41(28.9)	43(33.9)	41(33.3)	0.625§
Presence of pain [n(%)]	99(25.3)	31(28.1)	36(28.3)	32(26.0)	0.458§
Age (mean ± SD)	65.07 ± 16.86	64.38 ± 16.64	64.19 ± 16.40	66.78 ± 17.57	0.398*
Systolic BP (mean ± SD)	135.44 ± 18.96	135.24 ± 18.74	133.70 ± 18.26	137.45 ± 19.88	0.292*
Diastolic BP (mean ± SD)	81.37 ± 12.43	81.92 ± 11.42	80.40 ± 13.45	81.75 ± 12.50	0.562*
Heart rate (mean ± SD)	86.40 ± 17.86	91.56 ± 17.30	85.26 ± 14.8	81.62 ± 19.84	<0.001*

n- number; %-percentage; §- Chi-Square (significance: p<0.05)

*- ANOVA (significance: p<0.05)

SD- standard deviation; BP- blood pressure; N- number of observations.

### Patient participants

#### Blood Pressure (BP)

There was an effect of time on the participants’ systolic and diastolic blood pressures within all the three study groups (p<0.001), but no group effect (systolic BP: p = 0.32; diastolic BP: p = 0.92) and no group*time effect on the participants’ BP (Tables 3 and 4). Post hoc power analysis [mean overall systolic BP (control vs. partially-stocked), t-tests, effect size = 0.0414, ⍺ err prob = 0.05] revealed a power of 1-β err prob = 0.063 for the current trial. To achieve a power of 90%, a sample size of 12253 participants must be included in each group (total n = 24506). For control vs. fully-stocked the post hoc power analysis revealed a power of 1-β err prob = 0.133 for the current trial and to require a power of 90% a sample size of 1969 was necessary per group (total n = 3938).

**Table 3:** Descriptive analysis showing the blood pressure (systolic, diastolic) and heart rate of the participants in the different waiting area settings.

	Systolic blood pressure			Diastolic blood pressure			Heart rate		
	CG	PSA	FSA	Control	PSA	FSA	CG	PSA	FSA
**On arrival**									
N	142	126	123	142	126	121	142	126	123
Missing		1			1	2		1	
Minimum	98.33	86.67	81.00	54.33	40.00	51.00	54.00	50.33	47.33
Maximum	195.00	193.67	213.00	110.33	113.33	126.67	130.33	131.33	158.00
Mean	135.24	133.62	137.46	81.92	80.52	81.75	91.56	85.16	81.62
SD	18.74	18.31	19.88	11.42	13.45	12.50	17.30	14.84	19.84
**After 20 mins of waiting**									
Minimum	105.00	78.67	79.67	56.67	47.00	50.33	49.33	52.00	49.67
Maximum	199.33	175.00	197.33	109.00	108.33	124.33	132.00	132.67	146.67
Mean	131.79	130.19	133.41	79.63	79.21	80.09	88.56	83.17	79.68
SD	16.54	17.84	18.37	10.49	12.65	12.70	15.66	15.44	18.14

N- number; CG- Control group; PSA- Partially-stocked aquarium; FSA- Fully-stocked aquarium; SD- standard deviation.

**Table 4:** Differences in BP and heart rate of the participants in the different waiting area settings (ANOVA with repeated measures, significance p<0.05).

Source	Measure	Type III Sum of Squares	df	Mean Square	F	Sig.
**Time**	Systolic BP	2306.42	1	2306.42	44.82	<0.001
	Diastolic BP	511.90	1	511.90	25.10	<0.001
	Heart rate	1026.61	1	1026.61	40.94	<0.001
**Error (Time)**	Systolic BP	6174.99	120	51.46		
	Diastolic BP	2447.36	120	20.40		
	Heart rate	3009.46	120	25.08		
**Group**	Systolic BP	541.51	1	541.51	1.01	0.32
	Diastolic BP	3.26	1	3.26	0.01	0.92
	Heart rate	11879.91	1	11879.91	21.59	<0.001
**Error (Group)**	Systolic BP	64389.39	120	536.58		
	Diastolic BP	35214.83	120	293.46		
	Heart rate	66030.46	120	550.25		
Group * Time	Systolic BP	39.76	1	39.76	0.89	0.35
	Diastolic BP	5.65	1	5.65	0.31	0.58
	Heart rate	34.23	1	34.23	1.04	0.31
Error (Group*Time)	Systolic BP	5338.95	120	44.49		
	Diastolic BP	2194.43	120	18.29		
	Heart rate	3944.24	120	32.87		

BP- blood pressure; Sig.- significance (p<0.05); Valid N (listwise) = 121.

#### Heart Rate (HR)

There was an effect of time on the participants’ HR within all the three study groups (p<0.001), and a group effect (p<0.001); but no group*time effect (p = 0.31; Tables 3 and 4)

#### Anxiety and mood

There were no significant differences in the patients’ perception of their anxiety and mood scores between the study groups, as well as for the therapists’ evaluation of the patients’ anxiety levels (Tables 5 and 7).

**Table 5:** Descriptive analysis of the results of the anxiety, mood of the participants in the different waiting area settings.

**Anxiety**						
**6-Item State Trait Anxiety Inventory**	**Participant’s Subjective Assessment**			**Therapist’s Subjective Assessment**		
	**CG**	**PSA**	**FSA**	**CG**	**PSA**	**FSA**
N	142	127	123	142	127	122
Missing	-	-	-	-	-	1
Mean	10.18	10.37	9.81	11.23	10.93	10.87
Median	10.00	10.00	9.00	11.00	11.00	10.00
Mode	9.00	6.00	9.00	6.00	9.00	10.00
Standard deviation	3.12	3.33	3.51	3.46	3.45	3.36
Range	14.00	18.00	23.00	15.00	18.00	15.00
Minimum	6.00	6.00	1.00	6.00	6.00	6.00
Maximum	20.00	24.00	24.00	21.00	24.00	21.00
Percentiles						
25.00	8.00	8.00	7.00	9.00	9.00	8.00
50.00	10.00	10.00	9.00	11.00	11.00	10.00
75.00	12.00	12.00	12.00	14.00	13.00	13.00
**Mood**						
	**Feeling scale**			**Felt arousal scale**		
	**CG**	**PSA**	**FSA**	**CG**	**PSA**	**FSA**
N	142	127	122	142	127	122
Missing	-	-	1	-	-	1
Mean	1.92	2.24	2.04	2.07	1.87	2.01
Median	2.00	3.00	3.00	1.00	1.00	1.00
Mode	3.00	3.00	3.00	1.00	1.00	1.00
Standard deviation	1.95	2.16	2.04	1.37	1.28	1.39
Range	9.00	10.00	8.00	5.00	5.00	5.00
Minimum	-4.00	-5.00	-3.00	1.00	1.00	1.00
Maximum	5.00	5.00	5.00	6.00	6.00	6.00
Percentiles						
25.00	0.75	0.00	0.00	1.00	1.00	1.00
50.00	2.00	3.00	3.00	1.00	1.00	1.00
75.00	3.00	4.00	3.00	3.00	3.00	3.00

N- number; CG- Control group; PSA- Partially-stocked aquarium; FSA- Fully-stocked aquarium.

Post hoc power analysis for the patients’ perceived mean STAI-6 scores revealed a power of 0.077, and 0.147, for control vs. partially-stocked, and control vs. fully-stocked comparisons, respectively, in the current trial. To achieve a power of 90%, a sample size of 6062, and 1694 participants must be included in each group for control vs. partially-stocked, and control vs. fully-stocked comparisons, respectively. Post hoc power analysis for the therapists’ evaluation of the patients’ STAI-6 scores revealed a power of 0.109, and 0.137, for control vs. partially-stocked, and control vs. fully-stocked comparisons, respectively, in the current trial. To achieve a power of 90%, a sample size of 2789, and 1887 participants must be included in each group for control vs. partially-stocked, and control vs. fully-stocked comparisons, respectively. Post hoc power analysis for the FS scores revealed a power of 0.139, and 0.078, for control vs. partially-stocked, and control vs. fully-stocked comparisons, respectively, in the current trial. To achieve a power of 90%, a sample size of 862, and 5756 participants must be included in each group for control vs. partially-stocked, and control vs. fully-stocked comparisons, respectively. Post hoc power analysis for the FAS scores revealed a power of 0.234, and 0.064, for control vs. partially-stocked, and control vs. fully-stocked comparisons, respectively, in the current trial. To achieve a power of 90%, a sample size of 925, and 11119 participants must be included in each group for control vs. partially-stocked, and control vs. fully-stocked comparisons, respectively.

#### Patients’ subjective waiting area assessment

The participants from the FSA group rated their waiting room experience significantly higher than all the other groups (p<0.001, Tables 6 ànd 7).

**Table 6:** Descriptive analysis of the results of the participants’ subjective assessment of the different waiting room settings.

	CG	PSA	FSA
N	142	127	122
Missing	-	-	1
Mean	15.99	17.53	18.07
Median	16.00	18.00	18.00
Mode	16.00	16.00	21.00
Standard deviation	3.64	3.93	3.65
Range	20.00	24.00	16.00
Minimum	5.00	1.00	9.00
Maximum	25.00	25.00	25.00
Percentiles			
25.00	14.00	15.00	16.00
50.00	16.00	18.00	18.00
75.00	18.00	21.00	21.00

N- number; CG- Control group; PSA- Partially-stocked aquarium; FSA- Fully-stocked aquarium.

**Table 7:** Intergroup comparison of anxiety and mood of the participants in the different waiting area setups.

Outcome parameters	Groups	N	Mean Rank	Kruskal-Wallis H	df	p-value	>Median	< = Median	Median	Chi-Square	df	p-value*
STAI-6: Participant’s assessment	CG	142	200.60	2.76	2	0.25	73.00	69.00	9.50	4.82	2	0.09
	PSA	127	205.19				71.00	56.00				
	FSA	123	182.80				52.00	71.00				
	Total	392										
STAI-6: Therapist’s assessments	CG	142	204.08	1.16	2	0.56	67.00	75.00	11.00	1.64	2	0.44
	PSA	127	192.16				55.00	72.00				
	FSA	122	190.59				48.00	74.00				
	Total	391										
Feeling Scale Score	CG	142	187.98	2.28	2	0.32	24.00	118.00	3.00	3.14	2	0.21
	PSA	127	207.72				32.00	95.00				
	FSA	122	193.14				29.00	93.00				
	Total	391										
Felt Arousal Scale Score	CG	142	203.60	1.54	2	0.46	69.00	73.00	1.00	1.53	2	0.47
	PSA	127	188.08				53.00	74.00				
	FSA	122	195.40				52.00	70.00				
	Total	391										
Participants’ subjective assessment of the WA	CG	142	162.11	20.98	2	<0.001	51.00	91.00	17.00	13.66	2	0.001
	PSA	127	209.16				64.00	63.00				
	FSA	122	221.75				71.00	51.00				
	Total	391										

N- number; P-value: Kruskal-Wallis test, Significance: p<0.05; *- median test; STAI-6: 6- Item State Trait Anxiety Inventory; CG- Control group; PSA- Partially-stocked aquarium; FSA- Fully-stocked aquarium; SD- Standard deviation; WA- waiting area.

## Discussion

This controlled clinical trial found that there was an effect of waiting time on the BPs and heart rates of the participants (p<0.001), and no group*time effect. Furthermore, the study was not able to confirm an improvement in subjective anxiety assessments (STAI-6), and moods (FS and FAS) of the patients. Therefore, the null hypothesis is not rejected.

The study however, did reveal that the participants’ subjective assessment of the waiting area with the partial- and fully- stocked aquarium groups were better than the control waiting area. The effect of interacting with fish has the potential to influence the wellbeing in humans in a positive manner [11]. Reports suggest that maintaining an aquarium at home has been associated with relaxation [20, 21]. However, the current trial did not provide evidence for this. Our study findings have been observed in previous experiments where there were no effects of interaction with fish on the physiological and subjective anxiety measurements [11, 19, 22].

We believe that our results may have been influenced by several factors including lack of randomization, exposure time, concentration/focus, and age of the participants. The lack of randomization could be considered a big limitation of this study, and the comparisons of the groups may have been challenged. It is well known that in non-randomized comparisons the group comparisons must be matched by propensity scoring techniques and this was lacking in the current study; therefore, lacking a statistical control. Unfortunately, a propensity scoring is difficult in studies where there are more than two-groups and this study comprised of three groups. The participants in this study, as aforementioned, were sequentially recruited and once the sample number was achieved for the recruiting group, the participants were then included into the subsequent study groups. The first set of participants were enrolled into the control group, then into the partially-stocked aquarium group, and finally into the fully-stocked aquarium group. Although not randomised, the study was single-blinded as the participants were blinded to the exact intervention and the outcome. This does not eliminate the consequences of a lack of randomization, but nevertheless, still could be a factor that minimises the lack of it.

Exposure time or the time spent on viewing the aquarium could have been an important factor in influencing the results [3]. Studies on interaction with fish that have demonstrated a positive influence on the anxiety, stress, and health of participants, had considerably longer exposure times [8, 20, 21, 23]. Nevertheless, the 20 minutes exposure time fixed in this study could be considered acceptable, especially since previous studies with similar outcomes have used timeframes ranging between 20-40mins [7, 8, 22]. However, studies demonstrated positive effects even with shorter exposure times [3, 24]. In the experimental design of Cracknell et al. (2016) the participants were exposed to an aquarium exhibit and had to observe it for a specified time [3]. In our study, the participants were blinded and were not made aware to focus and observe solely on the aquarium. Instead, they were free to observe all aspects of the waiting area. In effect, in their 20 minutes of wait, their exposure time might have been considerably less to none. This factor could have played a significant role on their physiological and subjective stress measurements. Finally, the age of the participants seems to play a role in the outcomes. Studies with younger cohorts (mean age range: 14.2–37.1 years) tend to have a positive influence on the outcomes after interaction with fish and animals [3, 4, 7, 20, 21, 23–25]. Most studies with a middle-aged cohort tend to show no effect [26, 27]. However, with an age-advanced cohort with dementia, the effects were again relevant [8, 9, 28]. Our study had a mixed aged cohort and the mean age was 65.07 years; perhaps this could have been a factor in not demonstrating completely positive findings as seen in previous studies [19, 26, 27]. Another factor which may have influenced our results could have been the sample size. Although the required sample sizes were calculated prior to the start of the study, our results demonstrated that for some of the outcomes measured it had been underpowered. For example, post hoc power analysis for blood pressure revealed a required sample size of 12253 participants. Similarly, the study required around 5756 and 11119 participants, for FS and FAS respectively, for the control vs. fully-stocked comparisons. These numbers were clearly too high for us to recruit, both in a logistical as well as a financial aspect. Nevertheless, the participants did prefer the waiting area ambience with the partially-stocked/fully-stocked aquarium over the control. This does indicate that it did play a role on the participants mood although not significant and perhaps could have been significant, if the exposure times and the samples sizes were larger.

## Conclusions

Based on the findings of this trial, we conclude that the presence of an aquarium in the waiting area of a geriatric dental clinic did not seem to the participants’ blood pressure, heart rate, anxiety, or mood.

## Supporting information

S1 Checklist CONSORT 2010 checklist of information to include when reporting a randomised trial*. (PDF)

S1 File (PDF)

S1 Protocol (PDF)
