# Supplementary material for: Effect of the presence of an aquarium in the waiting area on the stress, anxiety and mood of adult dental patients: A controlled clinical trial
Source: PLoS One. 2021 Oct 12;16(10):e0258118. doi: 10.1371/journal.pone.0258118 (PMC8509982; doi:10.1371/journal.pone.0258118)
Supplement: S1 File — (PDF) [file pone.0258118.s002.pdf]

Kanton Zürich
**Kantonale Ethikkommission**

**Prof. Dr. med. Peter Meier-Abt**
Präsident

**Dr. med. Peter Kleist**
Geschäftsführer
Stampfenbachstrasse 121
Postfach
8090 Zürich
Telefon +41 43 259 79 70
Fax +41 43 259 79 72
admin.kek@kek.zh.ch
www.kek.zh.ch

Einschreiben
Universität Zürich
Zentrum für Zahnmedizin
Klinik für Allgemein-,
Behinderten- und Seniorenzahnmedizin
Prof. Dr.med. dent. Murali Srinivasan
Plattenstrasse 11
8032 Zürich

04. Juni 2020 / ktr

# Verfügung der Kantonalen Ethikkommission Zürich

| | |
|---|---|
| **BASEC-Nr.** | 2020-01315 |
| **Projekttitel** | Effect of the presence of an aquarium in the waiting room on the pre-treatment stress and anxiety levels of adult patients and reception staff |
| **Gesuchsteller/in** | Prof. Dr. med. dent. Murali Srinivasan, UZH, ZZM |
| **Zentrum** | Prof. Dr. med. dent. Murali Srinivasan, UZH, ZZM |

## Entscheid

**Auf das Gesuch wird nicht eingetreten.**
Begründung:

Das von Ihnen geplante Projekt fällt nicht in den Geltungsbereich des Humanforschungsgesetzes und bedarf deshalb für seine Durchführung keiner Bewilligung der Kantonalen Ethikkommission. Die Kantonale Ethikkommission ist somit für die Beurteilung des Projekts sachlich nicht zuständig.

## Empfehlungen bez. Durchführung

### Studienprotokoll

- Um die Verblindung zu gewährleisten, sollten Sie die Teilnehmenden in einem ersten Schritt nur **partiell** aufklären z.B. wird lediglich informiert, dass Patienten im Wartezimmer untersucht werden. Nach der Datenerhebung sollte im Debriefing darauf hingewiesen werden, dass es in erster Linie um das Aquarium (fehlend, mit und ohne Fische) ging. Die Teilnehmer haben dann die Wahl, ihr Einverständnis noch zurückzunehmen, ohne dass ihre Daten ausgewertet werden dürfen.
- Kapitel 4.3: Die Wartezeit sollte sich für die Teilnehmenden nicht verlängern. Zwischen den Messungen müssen 20 Minuten liegen…

**Patienten-Probanden-Information**

- Kapitel 4: Geben Sie noch an, wie hoch der zeitliche Aufwand einer Studienteilnahme ist.
- Patienten Zeile 42: Statt dem Geburtsdatum, sollte aus Datenschutzrechtlichen Gründen besser nur das Alter abgefragt werden.
- Kapitel 5: Die Puls- und Blutdruckmessung können Sie hier aufführen, Sie müssen aber auch dazuschreiben, dass die Studienteilnahme an sich keinen persönlichen Nutzen bringt.
- *staff-ICF* Kapitel 6: Geben Sie an, dass eine Nichtteilnahme oder ein Rückzug des Einverständnisses keine negativen Konsequenzen auf das Arbeitsverhältnis hat.
- Kapitel 11: Ergänzen Sie noch, dass die Daten anschliessend anonymisiert werden, d.h. dass nachher niemand mehr wissen kann, von wem die Daten ursprünglich stammten.
- Zeile 161f. (*staff* Zeile 142) Löschen Sie: "Ich akzeptiere, dass mein Arzt über meine Teilnahme an der Studie informiert wird."
- Zeile 169f. (*staff* Zeile 149f.) Lösche Sie: "Mir wurde mitgeteilt, dass eine Versicherung abgeschlossen wurde, um die Schäden abzudecken, die ich aufgrund der Studie erleiden könnte."

Kontaktperson: Dr. sc. nat. Tobias Rosenberger

## Entscheidverfahren

☐ ordentliches Verfahren ☐ vereinfachtes Verfahren ☒ Präsidialentscheid

Am Entscheid beteiligte Kommissionsmitglieder siehe Anhang.

Die Ethikkommission bestätigt, dass sie nach ICH-GCP arbeitet.

## Gebühren

Betrag: CHF 300.- Tarifcode: 6.0
Gemäss der geltenden Gebührenordnung von swissethics.

## Rechtsmittelbelehrung

Gegen diesen Beschluss kann innert 30 Tagen, von der Mitteilung an gerechnet, beim Regierungsrat des Kantons Zürich schriftlich Rekurs eingereicht werden. Die Rekursschrift muss einen Antrag und dessen Begründung enthalten. Der angefochtene Entscheid ist beizulegen oder genau zu bezeichnen. Die angerufenen Beweismittel sind genau zu bezeichnen und soweit möglich beizulegen.

**Kopie an**

- ☐ Sponsor
- ☐ Swissmedic
- ☐ Bundesamt für Gesundheit
- ☐ beteiligte Ethikkommission
- ☒ andere: Andrea Lundberg, Elena van der Heijden

Prof. Dr. med. Peter Meier-Abt
Präsident

Dr. med. Peter Kleist
Geschäftsführer

Anhang:
- Allgemeine Hinweise
- Eingereichte Dokumente
- Am Entscheid beteiligte Kommissionmitglieder der Abteilung A

**Allgemeine Hinweise:**

**Weitere Bewilligungspflichten:** Unterliegt das Forschungsvorhaben einer weiteren Bewilligungspflicht (Swissmedic/Bundesamt für Gesundheit), darf mit dessen Durchführung erst begonnen werden, wenn beide Bewilligungen vorliegen und diese auf identische Versionen der Gesuchsunterlagen beruhen. Stimmen die Versionen nicht überein, muss zur Angleichung der Dokumente eine Änderung eingereicht werden.

**Einreichung Dokumente**: Revidierte und neue Dokumente zum Forschungsvorhaben sollen ausschliesslich über das Web-Portal BASEC auf der entsprechenden Formularseite des betreffenden Gesuches eingereicht werden. Obsolete Dokumente sind zu entfernen und bei neuen oder angepassten Dokumenten sind Datums- und Versionsangaben entsprechend zu ergänzen. Die erfolgten Änderungen müssen im Korrekturmodus abgefasst und zusätzlich als Version ohne Markierungen eingereicht werden. Änderungen, die nicht markiert sind, werden nicht überprüft und sind nicht Gegenstand der Bewilligung. Die Studieninformationen und -einwilligungen, das Protokoll und die Änderungen müssen in MS Word- oder durchsuchbaren PDF-Dateien eingereicht werden, insbesondere müssen gescannte Dokumente eine Texterkennung durchlaufen haben (OCR). Die handschriftlich unterzeichneten Unterschriftenseiten sind in eingescannter Form einzureichen. Das unterschriebene und datierte Begleitschreiben muss die Antworten auf eventuell von der EK gestellte Fragen enthalten.

**Sprachversionen:** Die zuständige Ethikkommission überprüft im Rahmen des Bewilligungsverfahrens Unterlagen zur Rekrutierung, zur Aufklärung, zur Einwilligung und zur Erhebung von Daten in ihrer Amtssprache. Unterlagen in einer anderen Sprache werden von der Ethikkommission lediglich zur Kenntnis genommen. Für die korrekte Übersetzung ist der Sponsor oder die Projektleitung verantwortlich.

**Registrierung klinischer Versuche**: Der Sponsor ist verpflichtet, den klinischen Versuch in einem WHO-Primärregister oder im Register der Nationalen Medizinbibliothek der USA (clinicaltrials.gov) zu erfassen und anschliessend die Referenznummer des Registerbeitrags im BASEC-Portal unter dem Screen SNCTP einzugeben. Die Übertragung der erforderlichen Daten in das Swiss National Clinical Trials Portal (SNCTP) erfolgt nach Bewilligung der Ethikkommission und Zustimmung des Gesuchstellers automatisch. Die Informationen über den klinischen Versuch sind in beiden Registern öffentlich zugänglich.
Swissethics veröffentlicht zudem wenige Informationen wie Titel, Projekttyp oder Leit-Ethikkommission der durch die kantonalen Ethikkommissionen bewilligten Gesuche auf swissethics.ch (ausser klinische Phase-I-Arzneimittelversuche).

## Eingereichte Dokumente für das Hauptzentrum

**Professor Dr. Murali Srinivasan, Clinic of General, Special care and Geriatric Dentistry, Center of Dental Medicine, University of Zurich, Zurich**

| Dokument | Dok.Datum | Version |
|---|---|---|
| **1. Cover Letter** | | |
| 0-cover-letter-ec-lundberg-aquarium-study-copy.pdf | 18/05/2020 | |
| **2. Synopsis of the study plan** | | |
| 0-study-synopsis-uzh-abs-2020-1.pdf | 18/05/2020 | 1 |
| **3. Participant information sheet and informed consent (ICF)** | | |
| appendix-5-patient-consent-uzh-abs-2020-1.pdf | 18/05/2020 | 1 |
| appendix-6-receptionist-consent-uzh-abs-2020-1.pdf | 18/05/2020 | 1 |
| **4. Study plan (protocol), signed and dated** | | |
| 0-uzhabs20201-clinical-protocol-clino.pdf | 18/05/2020 | 1 |
| **6. Investigator's CV, dated** | | |
| appendix-17-cv-pi-pl-srinivasan.pdf | 17/05/2020 | |
| appendix-20-cv-co-i-lundberg.pdf | 18/05/2020 | |
| **10. Insurance** | | |
| see doc/cat: **6.8**, page/ref: **16** | | |
| **11. Other documents handed over to study participants** | | |
| No other documents handed over to study participants | | |
| **12. Details on nature and scope/value of compensation for participants** | | |
| There is no compensation for the participation in this study | | |
| **13. Other personnel** | | |
| appendix-14-staff-list-uzh-abs-2020-1.pdf | 18/05/2020 | |
| **14. Information on secure handling of biological material and personal data, and in particular on the storage thereof** | | |
| appendix-12-information-on-secure-handling-of-biological-material-uzh-abs-2020-01.pdf | 18/05/2020 | |
| **39. Miscellaneous / Varia** | | |
| appendix-9-details-on-nature-and-scope-of-the-compensation-for-participants-uzh-abs-2020-1-version-1.pdf | 18/05/2020 | 1 |
| appendix-15-signature-page.pdf | 18/05/2020 | 1 |
| appendix-16-cover-letter-ec-lundberg-aquarium-study.pdf | 18/05/2020 | 1 |
| appendix-7-schedule-of-assessment-uzh-abs-2020-01.pdf | 18/05/2020 | 1 |

| | | |
|---|---|---|
| appendix-1-study-summary-uzh-abs-2020-1.pdf | 18/05/2020 | 1 |
| appendix-2-stai-6-uzh-abs-2020-01.pdf | 18/05/2020 | 1 |
| appendix-3-feelingscale-arousalscale-uzh-abs-2020-01.pdf | 18/05/2020 | 1 |
| appendix-4-generalquestionaire-uzh-abs-2020-01.pdf | 18/05/2020 | 1 |

## Zusammensetzung der am Entscheid beteiligten Kommission

| Name, Vorname | am Entscheid beteiligt |
|---|---|
| Prof. Dr. med. Meier-Abt, Peter | ☒ |
| Dr. med. Bridler, René | ☐ |
| Dr. theol. Baumann-Hölzle, Ruth | ☐ |
| PD Dr. med. Betschart, Cornelia | ☐ |
| Prof. Dr. med. Bloch, Konrad E. | ☐ |
| PhD MNSc Geschwindner, Heike | ☐ |
| PD Dr.med. Dr.med.dent. Jacobsen, Christine | ☐ |
| Dr. Jeker, Raphael | ☐ |
| PD Dr. med. Jetter, Alexander | ☐ |
| Prof. Dr. rer. nat. Dipl.-Psych. Jokeit, Hennric | ☐ |
| Kapossy, Katrin, Fürsprecherin | ☐ |
| MScN Keller-Senn, Anita | ☐ |
| PD Dr. med. Künig, Gabriella | ☐ |
| Dr. med. Muff, Brigitte | ☐ |
| Prof. em. Dr. med. Lütolf, Urs M. | ☐ |
| Dr. iur. RA Mausbach, Julian | ☐ |
| Prof. Dr. med. Metzger, Urs | ☐ |
| Prof. Dr. med. Nadal, David | ☐ |
| Dr. med. dent. Ramel, Urs | ☐ |
| Prof. Rauch, Anita | ☐ |
| Prof. Dr. phil. Siegrist, Michael | ☐ |
| Prof. Dr. med. Spinas, Giatgen | ☐ |
| Prof. Dr. med. Stocker, Reto | ☐ |
| Lic. phil. Ziltener, Erika | ☐ |
