# Supplementary material for: Effect of the presence of an aquarium in the waiting area on the stress, anxiety and mood of adult dental patients: A controlled clinical trial
Source: PLoS One. 2021 Oct 12;16(10):e0258118. doi: 10.1371/journal.pone.0258118 (PMC8509982; doi:10.1371/journal.pone.0258118)
Supplement: S1 Protocol — (PDF) [file pone.0258118.s003.pdf]

# Clinical Protocol for ClinO, Chapter 4 "Other Clinical Trials"

## Project Title:

Effect of the presence of an aquarium in the waiting room on the pre-treatment stress and anxiety levels of adult patients and reception staff

## Project identifier:

UZH_ABS_2020_1

# Effect of the presence of an aquarium in the waiting room on the pre-treatment stress and anxiety levels of adult patients and reception staff

| | |
|---|---|
| Study Type: | Other Clinical Trial according to ClinO, Chapter 4 |
| Risk Categorisation: | Risk category A according to ClinO, Art. 61 |
| Study Registration: | It is planned to be registered in Clinical trials.gov |
| Sponsor-Investigator: | Professor Murali Srinivasan |
| Principal Investigator | Professor Murali Srinivasan,<br>Clinic director,<br>Clinic of General, Special care, and Geriatric dentistry,<br>Centre of Dental Medicine, University of Zurich,<br>Plattenstrasse 11, 8032 Zurich.<br>Tel no: +41 44 634 33 80; email: murali.srinivasan@zzm.uzh.ch |
| Investigated Intervention: | Effect of waiting room ambience |
| Protocol ID | UZH_ABS_2020_1 (internal id) |
| Version and Date: | Version 1 (dated 18/05/2020) |

**CONFIDENTIALITY STATEMENT**

The information contained in this document is confidential and the property of Professor Murali Srinivasan, Clinic of General, Special care, and Geriatric dentistry, Centre for Dental Medicine, University of Zurich, Zurich, Switzerland. The information may not in full or in part - be transmitted, reproduced, published, or disclosed to others than the applicable Competent Ethics Committee(s) and Regulatory Authority(ies) without prior written authorization from the principal investigator except to the extent necessary to obtain informed consent from those who will participate in the study.

## PROTOCOL SIGNATURE FORM

| | |
|---|---|
| Study Title | Effect of the presence of an aquarium in the waiting room on the pre-treatment stress and anxiety levels of adult patients and reception staff |
| Study ID | UZH_ABS_2020_1 (internal id) |

The Sponsor-Investigator [Principal Investigator (PI)] has approved the protocol version 1 (dated 18/05/2020) and confirm hereby to conduct the study according to the protocol, current version of the World Medical Association Declaration of Helsinki, and ICH-GCP guidelines as well as the local legally applicable requirements.

A clinical trial covered by ClinO Chapter 4 may be conducted in accordance with other rules than ICH-GCP guidelines, provided that such rules are recognised in the specialty in question and the protection of participants and data quality and security are guaranteed (ClinO Art. 5, Abs 2). If the clinical trial is not conducted according to ICH-GCP guidelines, the paragraph above must be adapted accordingly.

If Sponsor and Principal Investigator are the same person (Sponsor-Investigator), please delete the additional signature line for the Principal Investigator of the study.

**Sponsor-Investigator:**

Name: Professor Murali Srinivasan

Date: 18 May 2020 ______________ Signature: _

**Co-Investigator:**

Name: Ms. Andrea Lundberg

Date: 18 May 2020 ______________ Signature:

# TABLE OF CONTENTS


TABLE OF CONTENTS 4
GLOSSARY OF ABBREVATIONS 5
1 STUDY SYNOPSIS 6
2 BACKGROUND AND RATIONALE 10
3 STUDY OBJECTIVES AND DESIGN 11
3.1 Hypothesis and primary objective 11
3.2 Primary and secondary endpoints 11
3.3 Study design 12
3.4. Study intervention 12
4 STUDY POPULATION AND STUDY PROCEDURES 12
4.1 Inclusion and exclusion criteria, justification of study population 12
4.2 Recruitment, screening and informed consent procedure 13
4.3 Study procedures 13
4.4 Withdrawal and discontinuation 14
5 STATISTICS AND METHODOLOGY 14
5.1. Statistical analysis plan and sample size calculation 14
5.2. Handling of missing data and drop-outs 14
6 REGULATORY ASPECTS AND SAFETY 15
6.1 Local regulations / Declaration of Helsinki 15
6.2 (Serious) Adverse Events 15
6.3 (Periodic) safety reporting 16
6.4 Radiation 16
6.5 Pregnancy 16
6.6 Amendments 16
6.7 (Premature) termination of study 16
6.8 Insurance 16
7 FURTHER ASPECTS 17
7.1 Overall ethical considerations 17
7.2 Risk-benefit assessment 17
8 QUALITY CONTROL AND DATA PROTECTION 17
8.1 Quality measures 17
8.2 Data recording and source data 17
8.3 Confidentiality and coding 17
8.4 Retention and destruction of study data and biological material 18
9 MONITORIMG AND REGISTRATION 18
10 FUNDING / PUBLICATION / DECLARATION OF INTEREST 18
11 REFERENCES 19
12 APPENDIX LIST 21

## GLOSSARY OF ABBREVATIONS

| | |
|---|---|
| *AE* | *Adverse Event* |
| *ASR/DSUR* | *Annual Safety Repot / Development Safety Report* |
| *BASEC* | *Business Administration System for Ethical Committees* |
| *BP* | *Blood Pressure* |
| *Co-I* | *Co-Investigator* |
| *CRF* | *Case Report Form* |
| *CTCAE* | *Common Terminology Criteria for Adverse Events* |
| *FADP* | *Federal Act on Data Protection (in German: DSG, in French: LPD, in Italian: LPD)* |
| *eCRF* | *electronic Case Report Form* |
| *FAS* | *Felt Arousal Scale* |
| *FOPH* | *Federal Office of Public Health* |
| *FS* | *Feeling Scale* |
| *GCP* | *Good Clinical Practice* |
| *HR* | *Heart Rate* |
| *HRA* | *Human Research Act (in German: HFG, in French: LRH, in Italian: LRUm)* |
| *ICH* | *International Conference on Harmonisation* |
| *ClinO* | *Ordinance on Clinical Trials in Human Research (in German: KlinV, in French: OClin, in Italian: OSRUm)* |
| *QoL* | *Quality of Life* |
| *OHRQoL* | *Oral Health Related Quality of Life* |
| *PI* | *Principal Investigator* |
| *PL* | *Project Leader* |
| *SAE* | *Serious Adverse Event* |
| *SD* | *Standard Deviation* |
| *STAI* | *State-Trait Anxiety Inventory* |
| *STAI-6* | *Six-Item State-Trait Anxiety Inventory* |

# 1 STUDY SYNOPSIS

| | |
|---|---|
| **Sponsor / Sponsor-Investigator** | Professor Murali Srinivasan,<br>Clinic Director, Clinic of General, Special care and Geriatric Dentistry, |
| **Study Title** | Effect of the presence of an aquarium in the waiting room on the pre-treatment stress and anxiety levels of adult patients and reception staff |
| **Short Title / Study ID** | Lundberg Aquarium Study / UZH_ABS_2020_1 |
| **Protocol Version and Date** | Version 1 (dated 18/05/2020) |
| **Study Registration** | Not registered yet, It is planned to be registered in clinicaltrials.gov |
| **Study Category and Rationale** | Risk category A |
| **Background and Rationale** | Dental anxiety is a well-known, common dilemma affecting many adults, observed more frequently in children and women (1-7). It is an emotional apprehensive state which is multifactorial and the origin is most commonly attributed to the anticipation of the painful stimulus that may arise during treatment (8). The origin of this anxiety can be exogenic (environmental or prior traumatizing experiences), or endogenic (anxiety) (9, 10); other influential factors suggested are age, gender, level of education, frequency of dental treatment, cognitive ability, verbal intelligence, socio-economic status, and even iatrogenic factors (4, 11, 12). Dental anxiety may precipitate aversive behaviours, resulting in an avoidance in availing dental services and to advanced dental problems with complex treatment needs. This may cause further avoidance, and precipitate symptomatic sporadic treatment visits, which contribute to the further augmentation of the existing fear (13), deterioration of oral health (14, 15), affecting the quality of life (QoL) as well as the oral health related quality of life (OHRQoL) (16, 17).<br>Dental health professionals must take measures to treat and prevent this anxiety (18). It has been suggested that environmental factors affect/contribute to dental stress (19). Although quite trivial, the environment of a waiting room as well as the time spent waiting are parameters that can cause pre-treatment stress (20, 21). Therefore, the waiting room must be considered an important first port for dealing with the individual's pre-treatment dental anxiety. Therefore, it is cardinal that measures are taken from this point to distract the patient's apprehensions prior to the planned procedure. Evidence in literature suggest that waiting room ambience plays a significant role in reducing the patient anxiety (22). Exposure to images, audio-visual effects like the lighting, music and/or videos, presence of natural light, suitable reading material, aquariums have shown to positively influence patients prior to the dental treatment (23-30).<br>Beneficial effects of the presence of animals on the reduction of stress have been reported in literature (31, 32). Presence of a therapy dog on the patient's lap reduced the anxiety, stress and helped lower the pulse as well as the blood pressure during a dental procedure (31). The impact of the live fish has a positive effect on the psychological well-being and stress levels; higher relaxation, less anxiety and better mood was reported by participants after a period of observing live fish (33). Even observing videotapes of fish produced a increased relaxation in study participants when compared to control groups who watched only blank screens or videos of humans (34). Presence of aquariums generally tend to lower both systolic and diastolic blood pressures (33, 35, 36). Aquariums not only reduce stress, but interestingly increased pain thresholds in participants (37).<br>Although it has been evidenced that the dental anxiety and fear tends to decline with age it is imperative to understand that with advanced age, inherent age-associated problems exist. Cognitive decline, physical handicap, multimorbid status, care-resistant behaviour, general depression may all contribute to stress and may impact in the elders' cooperativeness for dental care. Aquariums in dementia units have demonstrated an improvement in the challenging behaviour, cooperativeness, and sleep of the residents as well as increase in the staff satisfaction (38). Residents in dementia units significantly also improved in their weights |

| | |
|---|---|
| | when an aquarium was introduced in their dining areas (39). Furthermore similar effects of reduction in blood pressure and anxiety even in the non-institutionalized elderly subjects (40). A recent systematic review concluded that although positive effects in terms of psychological and physiological wellbeing in humans have been evidenced when interacting with fish or aquariums, scientific evidence is still scarce and further research through well-designed studies with robust methodologies were deemed necessary (41). |
| **Risk / Benefit Assessment** | Not applicable. This is a category A study with minimum to no risk. There is no pharmacological or medical device being tested in this study. Only the effect of the waiting room ambience on the stress and anxiety of the participants is being tested. |
| **Objective(s)** | • The primary objective of this prospective study is to assess the effect of an aquarium present in the waiting area on the pre-treatment anxiety and stress levels of participants receiving dental care in a university setting geriatric dental clinic.<br>• The secondary objective is to evaluate the effect of an aquarium present in the waiting area on the stress and anxiety levels of the reception staff working in the geriatric dental clinic.<br>• The tertiary objective of this study was to verify the treating dentist's perception whether the waiting room ambience played any role in reducing the stress and anxiety levels of the participants. |
| **Endpoint(s)** | • **Heart rate (HR) and blood pressure (BP):** The pulse, heart rate and blood pressure will be measured of the participants upon their arrival into the dental clinic waiting area. Three measurements will be taken and then after 20 minutes of waiting another three measurements of the same will be made.<br>• **Anxiety:** Anxiety will be measured using a six-item State-Trait Anxiety Inventory (STAI-6) questionnaire: This questionnaire has six questions. Each of the questions has a 4-point Likert-scale response type (1- not at all, 2- somewhat, 3- moderately, 4- Very much). This will be given to the patient to be filled in before the patient will be called in for the scheduled dental treatment.<br>• **Mood (valence and arousal):** The mood of the participants was assessed for valence and arousal, using the feeling scale, and the felt arousal scale, questionnaires respectively. These two questionnaires will also be given to the participants to assess the valence and the arousal of the participants. The feeling scale measures the valence and has a scale between -5 (very bad) and +5 (very good). The felt arousal scale has a scale between 1 (low arousal) and 6 (high arousal). The participant has to just mark in the appropriate box of how they feel at the moment. The following secondary outcomes will be assessed in this study:<br>• Participants' subjective assessment of the waiting hall ambience and their waiting time: A purpose-made questionnaire with 5 questions will be administered to the participants during their waiting time, which will question their impressions of the ambience and time spent at the waiting room. Each of the questions has a 5-point Likert scale type response (1- strongly disagree, 2-somewhat disagree, 3- neutral, 4- somewhat agree, and 5- strongly agree) based on the questions the scores will be reversed whether it is a positively phrased or a negatively-phrased questions. Questions 1 and 2 are negatively phrased in this questionnaire.<br>• Clinician's assessment of the anxiety of the participant before the intended treatment: For the clinician's assessment the STAI-6 questionnaire, will be modified so that the questions will be directed towards the participant but answered by the clinician. |
| **Study Design** | This study is designed as a clinical trial that is non-randomized, three-arm, single-blinded, monocentric, regionally representative (Zurich). |
| **Statistical Considerations** | Data will be confirmed for a normal distribution using the K-S test. Mean and standard deviations will be calculated for BP, HR, PR, STAI-6, FS, FAS, and the subjective questionnaires. Intra- and inter- group differences will be calculated and ANOVA statistical models with post hoc tests will used be for statistical analysis with the significance level set to $\alpha=0.05$. All statistical analysis will be performed using a statistical software package (SPSS, version 25, IBM Corporation). |

| | |
|---|---|
| | The sample size was calculated using a freeware program (G*power 3.1.9.6 for Mac OS X) (42), from the significant results (mean and SD) from a previously published study of similar outcome pressure (33). For BP, the effect size was (dz=0.3241005) and the required sample size was calculated for α=0.05 and a power of 0.95 (1-β error probability) assuming a normal distribution. Therefore, the required total sample size was calculated as 126. Using the same study, the sample size for HR parameter was calculated using the effect size = 0.4881036 with a required sample size for α=0.05 and a power of 0.95 (1-β error probability) assuming a normal distribution, the required total sample size is 57. For valence, the effect size was 0.5771107 with α=0.05 and a power of 0.95 (1-β error probability) assuming a normal distribution, the total sample size required is 41. For the arousal, effect size was (dz=0.3241005) and the required sample size was calculated for α=0.05 and a power of 0.95 (1-β error probability) assuming a normal distribution, the calculated sample size is 30.<br><br>Therefore, based on the sample sizes calculated for the various parameters based on the previous published study, a sample size of 70 is fixed per participant group (groups: n=3, participants number: n=210) to avoid losses due to drop out and avoid type II statistical errors (43).<br><br>If the required sample size per group is not achieved in participant recruitment the trial will be terminated prematurely. |
| **Inclusion- / Exclusion Criteria** | **Inclusion criteria:**<br>- Adults ≥18 years<br>- Able to give informed consent as documented by signature.<br>**Exclusion criteria:**<br>- Inability to follow the procedures of the study, e.g. due to language problems, psychological disorders, dementia, etc.<br>- Visually impaired.<br>- Participants enrolled already in another clinical trial. |
| **Number of Participants with Rationale** | Total number of participants 210. Rationale explained above in the section statistical consideration. |
| **Study Intervention** | Effect presence of an aquarium in the waiting room on the Anxiety and stress levels of adult patients and reception staff in a geriatric clinic. |
| **Control Intervention** | Normal waiting room without an aquarium |
| **Study procedures** | The BP and HR will be checked for the participants during the waiting period prior to their dental procedure. The STAI-6, FS, and FAS questionnaires will be completed by the participants while they wait. |
| **Study Duration and Schedule** | **Study duration:** The study is planned for a duration of 3-6 months depending on the recruitment process. The recruitment will be consecutive, and once the required number for each study group is accomplished the recruitment for the subsequent group will commence. The study duration for each participant is the waiting time of the participant when the participant is waiting in the waiting room before being called in for the scheduled dental procedure. During the participant's waiting time, all the parameters required for the study will be measured and the questionnaires will be completed by the participants. The study ends once the participants exits the waiting room and proceeds to the planned dental procedure.<br><br>Planned 06/2020 of First-Participant-In<br>Planned 09/2020 of Last-Participant-Out |
| **Investigator(s)** | Professor Murali Srinivasan, Clinic Director, Clinic of General, Special care and Geriatric Dentistry, Centre of Dental Medicine, University of Zurich, Zurich Switzerland. Plattenstrasse 11, 8032 Zurich. Email: murali.srinivasan@zzm.uzh.ch<br><br>Ms. Andrea Lundberg, med. dent., Assistenzzahnärztin, Clinic of General, Special care and Geriatric Dentistry, Centre of Dental Medicine, University of Zurich, Zurich Switzerland. Plattenstrasse 11, 8032 Zurich. Email: andrea.lundberg@zzm.uzh.ch |

| | |
|---|---|
| **Study Center(s)** | Clinic of General, Special care and Geriatric Dentistry, Centre of Dental Medicine, University of Zurich, Zurich Switzerland. Plattenstrasse 11, 8032 Zurich. |
| **Data privacy** | Trial and participant data will be handled with uttermost discretion and is only accessible to authorised personnel who require the data to fulfil their duties within the scope of the study. On the CRFs and other study specific documents, participants are only identified by a unique participant number. Participant identification list will be stored by the principal investigator. It will be stored under lock and key. It will be protected from unauthorised or accidental disclosure, alteration, deletion, copying and theft. |
| **Ethical consideration** | The results can be generalized to the elderly population and patients with special needs. The scientific value would help improve the clinic waiting rooms where elderly patients and patients with special needs are involved. This would help improve the stress and anxiety of these special patients and hopefully help make them more cooperative and agreeable for dental treatment.<br>This is a category A study with minimal risks, the study evaluates the anxiety and stress of the participants waiting in the waiting room before they proceed to a dental procedure, only questionnaires are completed and measurement of the HR, BP and PR are the only clinical parameters recorded, therefore, no genetic data or biological samples will be collected. Moreover, the participation is completely voluntary and the participant can refuse participation without any consequence to the intended scheduled dental treatment. There will be no monetary or other forms of remuneration offered for participation in this study. However, the study ensures that there is will be an overall fair balance for the study participant. |
| **GCP Statement** | This study will be conducted in compliance with the protocol, the current version of the Declaration of Helsinki, the ICH-GCP, the HRA as well as other locally relevant legal and regulatory requirements. |

## 2 BACKGROUND AND RATIONALE

Dental anxiety is a well-known, common dilemma affecting many adults, observed more frequently in children and women (1-7). It is an emotional apprehensive state which is multifactorial and the origin is most commonly attributed to the anticipation of the painful stimulus that may arise during treatment (8). The origin of this anxiety can be exogenic (environmental or prior traumatizing experiences), or endogenic (anxiety) (9, 10); other influential factors suggested are age, gender, level of education, frequency of dental treatment, cognitive ability, verbal intelligence, socio-economic status, and even iatrogenic factors (4, 11, 12). Dental anxiety may precipitate aversive behaviours, resulting in an avoidance in availing dental services and to advanced dental problems with complex treatment needs. This may cause further avoidance, and precipitate symptomatic sporadic treatment visits, which contribute to the further augmentation of the existing fear (13), deterioration of oral health (14, 15), affecting the quality of life (QoL) as well as the oral health related quality of life (OHRQoL) (16, 17).

Dental health professionals must take measures to treat and prevent this anxiety (18). It has been suggested that environmental factors affect/contribute to dental stress (19). Although quite trivial, the environment of a waiting room as well as the time spent waiting are parameters that can cause pre-treatment stress (20, 21). Therefore, the waiting room must be considered an important first port for dealing with the individual's pre-treatment dental anxiety. Therefore, it is cardinal that measures are taken from this point to distract the patient's apprehensions prior to the planned procedure. Evidence in literature suggest that waiting room ambience plays a significant role in reducing the patient anxiety (22). Exposure to images, audio-visual effects like the lighting, music and/or videos, presence of natural light, suitable reading material, aquariums have shown to positively influence patients prior to the dental treatment (23-30).

Beneficial effects of the presence of animals on the reduction of stress have been reported in literature (31, 32). Presence of a therapy dog on the patient's lap reduced the anxiety, stress and helped lower the pulse as well as the blood pressure during a dental procedure (31). The impact of the live fish has a positive effect on the psychological well-being and stress levels; higher relaxation, less anxiety and better mood was reported by participants after a period of observing live fish (33). Even observing videotapes of fish produced a increased relaxation in study participants when compared to control groups who watched only blank screens or videos of humans (34). Presence of aquariums generally tend to lower both systolic and diastolic blood pressures (33, 35, 36). Aquariums not only reduce stress, but interestingly increased pain thresholds in participants (37).

Although it has been evidenced that the dental anxiety and fear tends to decline with age it is imperative to understand that with advanced age, inherent age-associated problems exist. Cognitive decline, physical handicap, multimorbid status, care-resistant behaviour, general depression may all contribute to stress and may impact in the elders' cooperativeness for dental care. Aquariums in dementia units have demonstrated an improvement in the challenging behaviour, cooperativeness, and sleep of the residents as well as increase in the staff satisfaction (38). Residents in dementia units significantly also improved in their weights when an aquarium was introduced in their dining areas (39). Furthermore similar effects of reduction in blood pressure and anxiety even in the non-institutionalized elderly subjects (40). A recent systematic review concluded that although positive effects in terms of psychological and physiological wellbeing in humans have been evidenced when interacting with fish or aquariums, scientific evidence is still scarce and further research through well-designed studies with robust methodologies were deemed necessary (41).

# 3 STUDY OBJECTIVES AND DESIGN

## 3.1 Hypothesis and primary objective

The hypotheses set for this study are:

- The primary null hypothesis is that there will be no effect of an aquarium present in the waiting area on the pre-treatment anxiety and stress levels of participants receiving dental care.
- The secondary hypothesis is that there will be no effect of an aquarium present in the waiting area on the stress and anxiety levels of the reception staff.
- The tertiary hypothesis for this study is that the treating dentist will not perceive any difference in the stress and anxiety levels of the participants after observing the aquarium in the waiting area.

**Objectives**

- The primary objective of this prospective study is to assess the effect of an aquarium present in the waiting area on the pre-treatment anxiety and stress levels of participants receiving dental care in a university setting geriatric dental clinic.
- The secondary objective is to evaluate the effect of an aquarium present in the waiting area on the stress and anxiety levels of the reception staff working in the geriatric dental clinic.
- The tertiary objective of this study was to verify the treating dentist's perception whether the waiting room ambience played any role in reducing the stress and anxiety levels of the participants.

## 3.2 Primary and secondary endpoints

**Primary endpoints:**

The primary endpoints/outcome measures assessed in this study are the stress and anxiety levels of the participants. The participants are subjected to a specific clinic waiting area ambience (1- no aquarium, 2- presence of aquarium with no fish, and 3- aquarium with fish) for their period of waiting before their intended dental visit and their stress and anxiety levels are measured.

The following parameters will be used to measure the stress and anxiety levels of the participants:

- **Heart rate (HR) and blood pressure (BP):** The pulse, heart rate and blood pressure will be measured of the participants upon their arrival into the dental clinic waiting area. Three measurements will be taken and then after 20 minutes of waiting another three measurements of the same will be made. BP and HR will not be recorded for the reception staff.
- **Anxiety:** Anxiety will be measured using a six-item State-Trait Anxiety Inventory (STAI-6) questionnaire (44): This questionnaire has six questions (Appendix 2). Each of the questions has a 4-point Likert-scale response type (1- not at all, 2- somewhat, 3- moderately, 4- Very much). This will be given to the patient to be filled in before the patient will be called in for the scheduled dental treatment.
- **Mood (valence and arousal):** The mood of the participants were assessed for valence and arousal, using the feeling scale (45), and the felt arousal scale (46), questionnaires respectively (Appendix 3). These two questionnaires will also be given to the participants to assess the valence and the arousal of the participants. The feeling scale measures the valence and has a scale between -5 (very bad) and +5 (very good). The felt arousal scale has a scale between 1 (low arousal) and 6 (high arousal). The participant has to just mark in the appropriate box of how they feel at the moment.

**Secondary endpoints:**

The following secondary outcomes will be assessed in this study:

- Participants' subjective assessment of the waiting hall ambience and their waiting time: A purpose-made questionnaire (Appendix 4) with 5 questions will be administered to the participants during their waiting time, which will question their impressions of the ambience and time spent at the waiting room. Each of the questions has a 5-point Likert scale type response (1- strongly disagree, 2-somewhat disagree, 3- neutral, 4- somewhat agree, and 5- strongly agree) based on the questions the scores will be reversed whether it is a positively phrased or a negatively-phrased questions. Questions 1 and 2 are negatively phrased in this questionnaire.
- Clinician's assessment of the anxiety of the participant before the intended treatment: For the clinician's assessment the STAI-6 questionnaire (45), will be modified so that the questions will directed towards the participant but answered by the clinician.

### 3.3 Study design

This study is designed as a clinical trial that is non-randomized, three-arm, single-blinded, monocentric, regionally representative (Zurich).

Methods of minimizing: Although the study is not randomized, it is a single-blinded study design. The participants are blinded to the intervention and the outcome. The participants will be sequentially allocated to the three intervention groups (1- no aquarium, 2- presence of aquarium with no fish, and 3- aquarium with fish). The participants will be given validated questionnaires to respond to the existing ambience of the waiting room and also report on their experience during the wait period.

### 3.4. Study intervention

The study intervention in this clinical study is the waiting room ambience, which the participants will be subjected to during their waiting time during their visit to the clinic of general, special care, and geriatric dentistry, at the centre of dental medicine, University of Zurich, Zurich Switzerland. The participants will be recruited into three of groups. The participants will be recruited into the three waiting room ambience groups.

Group 1 (Control): The participants in this group will be subjected to the normal waiting room without an aquarium present.

Group 2 (Intervention #1): These participants will be subjected to waiting in the same waiting room but with an aquarium present. There will be no fish present in the aquarium. The aquarium will be full equipped with all materials, water, etc., except the fish.

Group 3 (Intervention #2): These participants will be subjected to the waiting room with a fully equipped aquarium including the fish.

## 4 STUDY POPULATION AND STUDY PROCEDURES

### 4.1 Inclusion and exclusion criteria, justification of study population

The participants will be recruited from the patient pool who visit the Clinic for General, Special care and Geriatric dentistry in the Centre of Dental Medicine, University of Zurich, Zurich Switzerland. The patient are all elders and those patients with special needs, who require dental treatment.

**Inclusion criteria:**

- Adults ≥18 years
- Able to give informed consent as documented by signature.

**Exclusion criteria:**

- Inability to follow the procedures of the study, e.g. due to language problems,

psychological disorders, dementia, etc.
- Visually impaired.
- Participants enrolled already in another clinical trial.

## 4.2 Recruitment, screening and informed consent procedure

**Study location:** Participants were recruited form the patient pool who attend dental treatment visiting the Clinic for General, Special care and Geriatric dentistry in the Centre of Dental Medicine, University of Zurich, Zurich Switzerland. The reception staff who are employed in the clinic will be requested to participate.

**Participant recruitment:** Participants will consecutively through the co-investigator (Co-I) in daily clinical practice. No flyers or advertisements will be used for the recruitment process.

**Informed consent process (HRA, Art. 7, 16 - 18, 42; ClinO, Art. 7 - 9):** The investigators will explain to each participant the nature of the study, its purpose, the procedures involved, the expected duration, the potential risks and benefits and any discomfort it may entail. Each participant will be informed that the participation in the study is voluntary and that he or she may withdraw from the study at any time and that withdrawal of consent will not affect his or her subsequent dental assistance and treatment. The participant will be informed that his or her dental and/or medical records may be examined by authorised individuals other than their treating physician. All participants for the study will be provided a participant information sheet and a consent form describing the study and providing sufficient information for participant to make an informed decision about their participation in the study (Appendix 5 & 6). The participants will be requested for participation during their visit and sufficient time will be given to the participant to decide to participate or not. They will be given 15- 20 minutes to decide. The formal consent of a participant, using the approved consent form, will be obtained before the participant is submitted to any study procedure. The consent form will be signed and dated by the investigator or his designee at the same time as the participant sign. A copy of the signed informed consent will be given to the study participant. The consent form will be retained as part of the study records. The informed consent process will be documented in the patient file and any discrepancy to the process described in the protocol will be explained. The screening of the participants will be done based the predefined inclusion and exclusion criteria mentioned in section 4.1. Any screening procedure that is not routine or daily practice will only be performed once informed consent has been obtained.

No remuneration is offered to the participant.

## 4.3 Study procedures

**Study duration:** The study is planned for a duration of 3-6 months depending on the recruitment process. The recruitment will be consecutive, and once the required number for each study group is accomplished the recruitment for the subsequent group will commence.

The study duration for each participant is the waiting time of the participant when the participant is waiting in the waiting room before being called in for the scheduled dental procedure.

During the participant’s waiting time, all the parameters required for the study will be measured and the questionnaires will be completed by the participants. The study ends once the participants exits the waiting room and proceeds to the planned dental procedure.

A summary table listing all study visits, relevant procedures, and samplings as well as all timelines,

i.e. a schedule of assessment is shown in (Appendix 7).

### 4.4 Withdrawal and discontinuation

Participation in this trial is completely voluntary and the participant may choose to withdraw from this at any point without any consequence.

If the participant withdraws from the study before completing the necessary questionnaires or the measurements planned as explained in the pertaining to the protocol of the study, then a new participant will be recruited in place. Data and clinical measurements collected will not be used for analyses if the participant withdraws his consent or does not complete the measurements planned.

## 5 STATISTICS AND METHODOLOGY

### 5.1. Statistical analysis plan and sample size calculation

Data will be confirmed for a normal distribution using the K-S test. Mean and standard deviations will be calculated for BP, HR, STAI-6, FS, FAS, and the subjective questionnaires. Intra- and inter- group differences will be calculated and ANOVA statistical models with post hoc tests will used be for statistical analysis with the significance level set to α=0.05. All statistical analysis will be performed using a statistical software package (SPSS, version 25, IBM Corporation).

The sample size was calculated using a freeware program (G*power 3.1.9.6 for Mac OS X) (42), from the significant results (mean and SD) from a previously published study of similar outcome pressure (33). For BP, the effect size was (dz=0.3241005) and the required sample size was calculated for α=0.05 and a power of 0.95 (1-β error probability) assuming a normal distribution. Therefore, the required total sample size was calculated as 126. Using the same study, the sample size for HR parameter was calculated using the effect size = 0.4881036 with a required sample size for α=0.05 and a power of 0.95 (1-β error probability) assuming a normal distribution, the required total sample size is 57. For valence, the effect size was 0.5771107 with α=0.05 and a power of 0.95 (1-β error probability) assuming a normal distribution, the total sample size required is 41. For the arousal, effect size was (dz=0.3241005) and the required sample size was calculated for α=0.05 and a power of 0.95 (1-β error probability) assuming a normal distribution, the calculated sample size is 30.

Therefore, based on the sample sizes calculated for the various parameters based on the previous published study, a sample size of 70 is fixed per participant group (participant groups: n=3; Participant number: n=210) to avoid losses due to drop out and avoid type II statistical errors (43).

If the required sample size per group is not achieved in participant recruitment the trial will be terminated prematurely.

### 5.2. Handling of missing data and drop-outs

All data corresponding to drop-out participants (participant who do not complete the entire study) will be excluded from the final statistical analyses.

# 6 REGULATORY ASPECTS AND SAFETY

## 6.1 Local regulations / Declaration of Helsinki

This study is conducted in compliance with the protocol, the current version of the Declaration of Helsinki, the ICH-GCP, the HRA as well as other locally relevant legal and regulatory requirements.

## 6.2 (Serious) Adverse Events

An Adverse Event (AE) is any untoward medical occurrence in a patient or a clinical investigation subject which does not necessarily have a causal relationship with the trial procedure. An AE can therefore be any unfavourable or unintended finding, symptom, or disease temporally associated with a trial procedure, whether or not related to it.

A Serious Adverse Event (SAE) (ClinO, Art. 63) is any untoward medical occurrence that

- Results in death or is life-threatening,
- Requires in-patient hospitalisation or prolongation of existing hospitalisation,
- Results in persistent or significant disability or incapacity, or
- Causes a congenital anomaly or birth defect

Both PI and the Co-I will make a causality assessment of the event to the trial intervention. Any event assessed as possibly, probably or definitely related is classified as related to the trial intervention.

| Relationship | Description |
|---|---|
| Definitely | Temporal relationship<br>Improvement after dechallenge*<br>Recurrence after rechallenge<br>(or other proof of drug cause) |
| Probably | Temporal relationship<br>Improvement after dechallenge<br>No other cause evident |
| Possibly | Temporal relationship<br>Other cause possible |
| Unlikely | Any assessable reaction that does not fulfil the above conditions |
| Not related | Causal relationship can be ruled out |
| *Improvement after dechallenge only taken into consideration, if applicable to reaction | |

Both PI and Co-I will make a severity assessment of the event as mild, moderate or severe. Mild means the complication is tolerable, moderate means it interferes with daily activities and severe means it renders daily activities impossible.

**Reporting of SAEs** (ClinO, Art. 63)

All SAEs will be documented and reported immediately (within a maximum of 24 hours) to the Sponsor-Investigator of the study. If it cannot be excluded that the SAE occurring in Switzerland is attributable to the intervention under investigation, the PI and the Co-I will report it to the Ethics Committee via BASEC within 15 days.

### Follow up of (Serious) Adverse Events

Not applicable, in this study.

### 6.3 (Periodic) safety reporting

An annual safety report (ASR/DSUR) is submitted once a year to the local Ethics Committee by the Investigator (ClinO, Art. 43 Abs).

### 6.4 Radiation

Not applicable in this trial.

### 6.5 Pregnancy

Not applicable in this trial.

### 6.6 Amendments

Substantial changes to the study setup and study organization, the protocol and relevant study documents will be submitted to the Ethics Committee for approval before implementation. Under emergency circumstances, deviations from the protocol to protect the rights, safety and well-being of human subjects may proceed without prior approval of the Ethics Committee. Such deviations shall be documented and reported to the Ethics Committee as soon as possible. Substantial amendments are changes that affect the safety, health, rights and obligations of participants, changes in the protocol that affect study objective(s) or central research topic, changes of study site(s) or of study leader and sponsor (ClinO, Art. 29). A list of substantial changes will also be available on www.swissethics.ch. A list of all non-substantial amendments will be submitted once a year to the competent EC together with the ASR.

### 6.7 (Premature) termination of study

The Investigator may terminate the study prematurely according to certain circumstances, e.g.

- Ethical concerns,
- Insufficient participant recruitment,
- When the safety of the participants is doubtful or at risk (e.g. when the benefit-risk assessment is no longer positive),
- Alterations in accepted clinical practice that make the continuation of the study unwise, or
- Early evidence of harm or benefit of the experimental intervention

Upon regular study termination, the Ethics Committee will be notified via BASEC within 90 days (ClinO, Art. 38).

Upon premature study termination or study interruption, the Ethics Committee is notified via BASEC within 15 days (ClinO, Art. 38).

### 6.8 Insurance

Since this is a category A study, in the event of study-related damage or injuries, the liability of the institution, Centre of Dental Medicine, University of Zurich provides compensation, except for claims that arise from misconduct or gross negligence (Appendix 8).

# 7 FURTHER ASPECTS

## 7.1 Overall ethical considerations

The results can be generalized to the elderly population and patients with special needs. The scientific value would help improve the clinic waiting rooms where elderly patients and patients with special needs are involved. This would help improve the stress and anxiety of these special patients and hopefully help make them more cooperative and agreeable for dental treatment. This is a category A study with minimal risks, the study evaluates the anxiety and stress of the participants waiting in the waiting room before they proceed to a dental procedure, only questionnaires are completed and measurement of the HR, BP and PR are the only clinical parameters recorded, therefore, no genetic data or biological samples will be collected. Moreover, Participation is completely voluntary and the participant can refuse participation without any consequence to the intended scheduled dental treatment. There will be no monetary or other forms of remuneration offered for participation in this study. However, the study ensures that there is will be an overall fair balance for the study participant (Appendix 9).

## 7.2 Risk-benefit assessment

Not applicable. This is a category A study with minimum to no risk. There is no pharmacological or medical device being tested in this study. Only the effect of the waiting room ambience on the stress and anxiety of the participants is being tested.

# 8 QUALITY CONTROL AND DATA PROTECTION

## 8.1 Quality measures

All measures of quality assurance and quality control will be monitored. Measures to avoid double data entry will be undertaken. Study personnel are trained on all important study related aspects. A trial monitor will check the progress of the study and the data managed through planned quality visits. For quality assurance, the Ethics Committee or an independent trial monitor may visit the research sites. Direct access to the source data and all study related files is granted on such occasions. All involved parties keep the participant data strictly confidential.

## 8.2 Data recording and source data

Data will be recorded on paper case report form (CRF). An audit trail will be performed by the study trial monitor. For each participant a CRF is maintained (Appendix 10, & 11). CRFs will not identify participants by their name or birth date, but will provide appropriate coded identification. The source data will be obtained from participant’s dental and medical records, certified copies of original records of clinical findings, questionnaires, observations, or other recorded activities in the clinical investigation. Source data collected on study specific documents (e.g. study CRF, study specific forms or questionnaires, etc.), will be clearly differentiated from routinely collected data during the daily practice.

## 8.3 Confidentiality and coding

Trial and participant data will be handled with uttermost discretion and is only accessible to authorised personnel who require the data to fulfil their duties within the scope of the study. On the CRFs and other study specific documents, participants are only identified by a unique participant number.

Participant identification list will be stored by the principal investigator. It will be stored under lock and key. It will be protected from unauthorised or accidental disclosure, alteration, deletion, copying and theft.

There will be no biological material involved or collected in this study (Appendix 12).

### 8.4 Retention and destruction of study data and biological material

All study data will be archived for 10 years after study termination or premature termination of the study.

## 9 MONITORING AND REGISTRATION

A trial monitor within the centre of dental medicine will be appointed to perform timely monitoring and evaluate the progress of the study and ensure that all protocols are being followed per the study protocol as well as all the documents are being maintained, as stipulated (Appendix 13). The source data/documents will be accessible to study monitors and questions will be answered during monitoring. Registration in a national language in the Swiss National Clinical trial Portal (SNCTP via BASEC) will be done. In addition, the study will be registered in a registry listed in the WHO International Clinical Trials Registry Platform (ICTRP; http://www.who.int/ictrp/en/), if it satisfies the definition given therein.

## 10. FUNDING / PUBLICATION / DECLARATION OF INTEREST

No external funding will be received for the conception, execution and completion of this trial. All funding is from divisional funds allocated by the university of Zurich to the Clinic of general, special care and Geriatric dentistry at the Centre of Dental Medicine, University of Zurich.

The result of this study is planned to be published in a peer-reviewed dental journal in the domain of Gerodontology or Special care dentistry.

The PI, Co-I, and all involved in this study have no conflicts of interests related to this study. All involved in this study are employees of the Clinic of General, Special care and Geriatric Dentistry, Centre of Dental Medicine, University of Zurich

## 11. REFERENCES

1. Corah NL. Development of a dental anxiety scale. J Dent Res. 1969;48(4):596.
2. Corah NL, Pantera RE. Controlled study of psychologic stress in a dental procedure. J Dent Res. 1968;47(1):154-7.
3. Hakeberg M, Berggren U, Carlsson SG. Prevalence of dental anxiety in an adult population in a major urban area in Sweden. Community Dent Oral Epidemiol. 1992;20(2):97-101.
4. Armfield JM, Spencer AJ, Stewart JF. Dental fear in Australia: who's afraid of the dentist? Aust Dent J. 2006;51(1):78-85.
5. Liinavuori A, Tolvanen M, Pohjola V, Lahti S. Changes in dental fear among Finnish adults: a national survey. Community Dent Oral Epidemiol. 2016;44(2):128-34.
6. Smith TA, Heaton LJ. Fear of dental care: are we making any progress? J Am Dent Assoc. 2003;134(8):1101-8.
7. Folayan MO, Idehen EE, Ojo OO. The modulating effect of culture on the expression of dental anxiety in children: a literature review. Int J Paediatr Dent. 2004;14(4):241-5.
8. Armfield JM. How do we measure dental fear and what are we measuring anyway? Oral Health Prev Dent. 2010;8(2):107-15.
9. Weiner AA, Sheehan DV. Etiology of dental anxiety: psychological trauma or CNS chemical imbalance? Gen Dent. 1990;38(1):39-43.
10. Zinke A, Hannig C, Berth H. Psychological distress and anxiety compared amongst dental patients- results of a cross-sectional study in 1549 adults. BMC Oral Health. 2019;19(1):27.
11. Milgrom P, Newton JT, Boyle C, Heaton LJ, Donaldson N. The effects of dental anxiety and irregular attendance on referral for dental treatment under sedation within the National Health Service in London. Community Dent Oral Epidemiol. 2010;38(5):453-9.
12. Beaton L, Freeman R, Humphris G. Why are people afraid of the dentist? Observations and explanations. Med Princ Pract. 2014;23(4):295-301.
13. Armfield JM, Stewart JF, Spencer AJ. The vicious cycle of dental fear: exploring the interplay between oral health, service utilization and dental fear. BMC Oral Health. 2007;7:1.
14. Thomson WM, Stewart JF, Carter KD, Spencer AJ. Dental anxiety among Australians. Int Dent J. 1996;46(4):320-4.
15. Boyle CA, Newton T, Milgrom P. Who is referred for sedation for dentistry and why? Br Dent J. 2009;206(6):E12; discussion 322-3.
16. McGrath C, Bedi R. Measuring the impact of oral health on quality of life in Britain using OHQoL-UK(W). J Public Health Dent. 2003;63(2):73-7.
17. McGrath C, Bedi R. The association between dental anxiety and oral health-related quality of life in Britain. Community Dent Oral Epidemiol. 2004;32(1):67-72.
18. Liinavuori A, Tolvanen M, Pohjola V, Lahti S. Longitudinal interrelationships between dental fear and dental attendance among adult Finns in 2000-2011. Community Dent Oral Epidemiol. 2019;47(4):309-15.
19. Eli I, Uziel N, Baht R, Kleinhauz M. Antecedents of dental anxiety: learned responses versus personality traits. Community Dent Oral Epidemiol. 1997;25(3):233-7.
20. Coffey PA, Di Giusto J. The effects of waiting time and waiting room environment on dental patients' anxiety. Aust Dent J. 1983;28(3):139-42.
21. Peretz B, Efrat J. Dental anxiety among young adolescent patients in Israel. Int J Paediatr Dent. 2000;10(2):126-32.
22. Fux-Noy A, Zohar M, Herzog K, Shmueli A, Halperson E, Moskovitz M, et al. The effect of the waiting room's environment on level of anxiety experienced by children prior to dental treatment: a case control study. BMC Oral Health. 2019;19(1):294.
23. Bradt J, Dileo C, Shim M. Music interventions for preoperative anxiety. Cochrane Database Syst Rev. 2013(6):CD006908.
24. Gee NR, Reed T, Whiting A, Friedmann E, Snellgrove D, Sloman KA. Observing Live Fish Improves Perceptions of Mood, Relaxation and Anxiety, But Does Not Consistently Alter Heart Rate or Heart Rate Variability. Int J Environ Res Public Health. 2019;16(17).

25. Hasheminia D, Kalantar Motamedi MR, Karimi Ahmadabadi F, Hashemzehi H, Haghighat A. Can ambient orange fragrance reduce patient anxiety during surgical removal of impacted mandibular third molars? J Oral Maxillofac Surg. 2014;72(9):1671-6.
26. Kritsidima M, Newton T, Asimakopoulou K. The effects of lavender scent on dental patient anxiety levels: a cluster randomised-controlled trial. Community Dent Oral Epidemiol. 2010;38(1):83-7.
27. Benedetti F, Colombo C, Barbini B, Campori E, Smeraldi E. Morning sunlight reduces length of hospitalization in bipolar depression. J Affect Disord. 2001;62(3):221-3.
28. Choi SH, Won JH, Cha JY, Hwang CJ. Effect of Audiovisual Treatment Information on Relieving Anxiety in Patients Undergoing Impacted Mandibular Third Molar Removal. J Oral Maxillofac Surg. 2015;73(11):2087-92.
29. Lahti S, Suominen A, Freeman R, Lahteenoja T, Humphris G. Virtual Reality Relaxation to Decrease Dental Anxiety: Immediate Effect Randomized Clinical Trial. JDR Clin Trans Res. 2020:2380084420901679.
30. Fox C, Newton JT. A controlled trial of the impact of exposure to positive images of dentistry on anticipatory dental fear in children. Community Dent Oral Epidemiol. 2006;34(6):455-9.
31. Cruz-Fierro N, Vanegas-Farfano M, Gonzalez-Ramirez MT. Dog-Assisted Therapy and Dental Anxiety: A Pilot Study. Animals (Basel). 2019;9(8).
32. Kline JA, Fisher MA, Pettit KL, Linville CT, Beck AM. Controlled clinical trial of canine therapy versus usual care to reduce patient anxiety in the emergency department. PLoS One. 2019;14(1):e0209232.
33. Cracknell D, White MP, Pahl S, Nichols WJ, Depledge MH. Marine Biota and Psychological Well-Being: A Preliminary Examination of Dose-Response Effects in an Aquarium Setting. Environ Behav. 2016;48(10):1242-69.
34. Wells DL. The effect of videotapes of animals on cardiovascular responses to stress. Stress and Health. 2005;21(3):209-13.
35. Friedmann E, Sue AT, Son H, Chapa D, McCune M. Pet's Presence and Owner's Blood Pressures during the Daily Lives of Pet Owners with Pre- to Mild Hypertension. Anthrozoös. 2013;26(4):535-50.
36. Allen K, Shykoff BE, Izzo JL, Jr. Pet ownership, but not ace inhibitor therapy, blunts home blood pressure responses to mental stress. Hypertension. 2001;38(4):815-20.
37. Sanchez M, Delpont M, Bachy M, Kabbaj R, Annequin D, Vialle R. How can surgeonfish help pediatric surgeons? A pilot study investigating the antinociceptive effect of fish aquariums in adult volunteers. Pain Res Manag. 2015;20(1):e28-32.
38. Edwards NE, Beck AM, Lim E. Influence of aquariums on resident behavior and staff satisfaction in dementia units. West J Nurs Res. 2014;36(10):1309-22.
39. Edwards NE, Beck AM. The influence of aquariums on weight in individuals with dementia. Alzheimer Dis Assoc Disord. 2013;27(4):379-83.
40. Riddick CC. Health, aquariums, and the non-institutionalised elderly. Marriage and family review. 1985;8(3-4):163-73.
41. Clements H, Valentin S, Jenkins N, Rankin J, Baker JS, Gee N, et al. The effects of interacting with fish in aquariums on human health and well-being: A systematic review. PLoS One. 2019;14(7):e0220524.
42. Faul F, Erdfelder E, Buchner A, Lang AG. Statistical power analyses using G*Power 3.1: tests for correlation and regression analyses. Behavior research methods. 2009;41(4):1149-60.
43. Harrell FE, Jr., Lee KL, Califf RM, Pryor DB, Rosati RA. Regression modelling strategies for improved prognostic prediction. Statistics in medicine. 1984;3(2):143-52.
44. Marteau TM, Bekker H. The development of a six-item short-form of the state scale of the Spielberger State-Trait Anxiety Inventory (STAI). Br J Clin Psychol. 1992;31(3):301-6.
45. Hardy CJ, Rejeski WJ. Not what, but how one feels: the measurement of affect during exercise. . J Sport Exerc Psychol. 1989;11:304-17.
46. Svebak S, Murgatroyd S. Metamotivational dominance: a multimethod validation of reversal theory constructs. Journal of perception and social psychology. 1985;48:107-16.

## 12. APPENDIX LIST

1. Study Summary
2. STAI-6 questionnaire
3. FS & FAS questionnaire
4. Generalised questionnaire
5. Patient information and consent form
6. Reception staff information and consent form
7. Schedule of assessment
8. Insurance
9. Details on the nature of compensation of participants
10. CRF_ patients
11. CRF_receptionists
12. Information on secure handling of biological materials.
13. Monitoring plan
14. Staff list
15. Signature page
16. Covering letter from project leader (PI)
17. CV of PI/PL
18. PI’s proof of GCP training
19. PI’s proof of update of GCP training
20. CV of Co-I
21. Co-I’s proof of GCP training

# Bewertung der Auswirkung der Präsenz eines Aquariums im Wartebereich auf ältere Patienten und Empfangspersonal

Diese Studie wird von der Klinik für Allgemein-, Behinderten- und Seniorenzahnmedizin (ABS), am Zentrum für Zahnmedizin in Zürich (ZZM) der Universität Zürich (UZH), in Zürich, Schweiz, organisiert.

Geschätzte Dame, geschätzter Herr,

Wir laden Sie ein, an unserem Forschungsprojekt teilzunehmen. Dieses Informationsblatt beschreibt unser Forschungsprojekt.

## 1. Ziele der Studie

Ziel dieser Studie ist es, die Auswirkung der Präsenz eines Aquariums im Wartebereich unserer Klinik auf ältere Patienten und Empfangspersonal zu bewerten. Zu diesem Zweck nehmen Sie an einer Umfrage mittels Fragebogen teil. Außerdem werden bei Ihnen Puls und Blutdruck gemessen, und Ihr Gemütszustand wird von einer Fachperson eingeschätzt.

## 2. Auswahl der Personen, die zur Teilnahme an der Studie berechtigt sind

Die Teilnahme steht allen Personen offen, die sich an der Klinik für Alters-, Behinderten, und Seniorenzahnmedizin für eine ärztliche Besprechung oder Behandlung einfinden und gewillt sind, an unserer Studie teil zunehmen.

## 3. Allgemeine Informationen zur Studie

- Diese Studie dient der Bewertung unseres neuen Aquariums im Wartebereich der ABS Klinik.
- Sie werden gebeten, uns einige Angaben zu Ihrer Person zu machen.
- Anschließend nimmt eine Fachperson je drei Messungen von Puls und Blutdruck vor, und es werden Ihnen mehrere Fragen zu Ihrem Gemütszustand gestellt.
- Wir führen diese Studie in Übereinstimmung mit den Anforderungen der Schweizer Gesetzgebung durch.
- Wir befolgen alle international anerkannten Richtlinien.

## 4. Ablauf für die Teilnehmer

Falls Sie sich zur Teilnahme entscheiden ist der Ablauf der Studie folgendermaßen strukturiert:

- Bevor Studienbeginn geben wir Ihnen Informationen zur Teilnahme an der Studie.
- Entschließen Sie sich zur Teilnahme an der Studie bitten wir Sie uns einige Angaben zu Ihrer Person zu machen: Geburtsdatum, aktuelle Wohnsituation, körperliche Beeinträchtigungen, Pflegebedürftigkeit, Bluthochdruck, Medikamente, Schmerzen.
- Anschließend geben Sie uns an, welches Ambiente Sie im Wartebereich unserer Klinik vorfinden.
- Bei Ankunft und nach 20 Minuten nimmt eine Fachperson je drei Messungen

Ihres Pulses und Ihres Blutdruckes. Zur Information bezüglich Ihrer Privatsphäre werden Sie darauf hingewiesen, dass die Messungen im Wartebereich vorgenommen werden.

- Wir bitten Sie dann, uns Ihren Gemütszustand, sowie Ihre allgemeine Erregtheit, mittels der zur Verfügung gestellten Fragebögen mitzuteilen.
- Zum Schluss wird eine Fachperson nochmals Ihren Gemütszustand beurteilen.

**5. Vorteile für die Teilnehmer**

Durch Ihre Teilnahme an dieser Studie profitieren Sie von einer Puls- und Blutdruckmessung. Die Teilnahme an der Studie ist kostenlos.

**6. Rechte der Teilnehmer**

Sie nehmen nach eigenem Ermessen an dieser Studie teil. Wenn Sie sich dafür entscheiden, nicht teilzunehmen, oder wenn Sie sich dazu entschließen, Ihre Entscheidung im Verlauf der Studie zu überdenken, müssen Sie Ihre Ablehnung nicht begründen. Sie können jederzeit alle Fragen stellen, die für Ihr Verständnis der Studie erforderlich sind. Bitte wenden Sie sich an die Person, die am Ende dieses Informationsblatts angegeben ist.

**7. Pflichten der Teilnehmer**

Als Teilnehmer an der Studie erlauben Sie:

- Durchführung einer Puls- und Blutdruckmessung durch eine Fachperson.
- Auskunft über Ihren aktuellen Gemütszustand, mittels Frageboden zu geben.

**8. Risiken und Einschränkungen für die Teilnehmer**

Die Studie ist nicht invasiv und birgt kein besonderes Risiko. Aus Gründen der Privatsphäre, werden Sie darauf hingewiesen, dass sowohl alle medizinischen Messungen, sowohl die Befragungen im Wartezimmer stattfinden.

**9. Erkenntnisse während der Studie**

Der Prüfer wird Sie während der Studie über alle neuen Erkenntnisse informieren, die den Nutzen der Studie oder Ihre Sicherheit beeinträchtigen können, und daher Ihre Zustimmung zur Teilnahme. Sie werden mündlich und schriftlich informiert.

**10. Vertraulichkeit von Daten und Proben**

Zu Forschungszwecken gesammelte Daten werden während der Sammlung verschlüsselt. Codierung bedeutet, dass alle Daten, die Sie identifizieren (z. B. Name, Geburtsdatum usw.), durch einen Code ersetzt werden, sodass Personen, die diesen Code nicht kennen, diese Daten nicht mit einer anderen Person verknüpfen können. Der Code bleibt dauerhaft am Zentrum für Zahnmedizin. Alle an der Studie in irgendeiner Weise beteiligten Personen sind zur Wahrung des Berufsgeheimnisses verpflichtet. Wir garantieren die Einhaltung aller Datenschutzrichtlinien und zeigen Ihren Namen in keinem Print- oder Online-Bericht oder in keiner Veröffentlichung an. Sie haben jederzeit das Recht, Ihre Daten einzusehen.

## 11. Rücktritt vom Studium

Sie können jederzeit von der Studie zurücktreten, wenn Sie dies wünschen. Die bisher gesammelten Daten werden weiterhin analysiert, um den Wert der Studie insgesamt nicht zu beeinträchtigen.

## 12. Entschädigung der Teilnehmer

Wenn Sie an der Studie teilnehmen, profitieren Sie von einer Puls- sowie Blutdruckmessung. Wenn eine Behandlung erforderlich ist, helfen wir Ihnen, Kontakt mit einem Arzt Ihrer Wahl aufzunehmen. Ihre Teilnahme hat keine finanziellen Konsequenzen für Sie oder Ihre Krankenversicherung.

## 13. Finanzierung der Studie

Die Studie wird vollständig aus Mitteln finanziert, die der Klinik für Allgemein, Behinderten- und Seniorenzahnmedizin, Zentrum für Zahnmedizin, Zürich Schweiz, gewährt wurden.

## 14. Kontakt (e)

Im Zweifelsfall, bei Ängsten oder Notfällen während oder nach dem Studium können Sie sich jederzeit an eine der folgenden Personen wenden:

Untersuchungszahnärzte: med. dent. Andrea Lundberg, med. dent. Nicole Kalberer.

## 15. Einverständniserklärung

Schriftliche Einverständniserklärung zur Teilnahme an einem Forschungsprojekt.
Bitte lesen Sie dieses Formular sorgfältig durch. Sie können gerne Fragen stellen, wenn Sie diese nicht verstehen oder eine Erklärung wünschen.

**Titel der Studie:**
Bewertung der Auswirkung der Präsenz eines Aquariums im Wartebereich auf ältere Patienten und Empfangspersonal

**Verantwortliche Institution:**
Klinik für Allgemein-, Behinderten- und Seniorenzahnmedizin (ABS)
Zentrum für Zahnmedizin (ZZM)
Universität Zürich (UZH)
Plattenstrasse 11
CH-8032 Zürich | Schweiz

**Abteilung von**
Professor Murali SRINIVASAN
E-Mail: Murali.Srinivasan@zzm.uzh.ch

**Für das Projekt an Ort verantwortliche Zahnärzte:**
Med. dent. Andrea Lundberg, med. dent. Nicole Kalberer

**Teilnehmer / Teilnehmer:**

______________________________________________________

(Vor- und Nachname in Druckbuchstaben)

**Geburtsdatum:______________________** **O Frau O Mann**

- Ich erkläre, von dem für diese Studie verantwortlichen Prüfer/Zahnarzt mündlich oder schriftlich über die Ziele und den Verlauf der Studie sowie über die Auswirkungen, Vorteile, möglichen Nachteile und potenziellen Risiken informiert worden zu sein.
- Ich nehme freiwillig an dieser Studie teil und akzeptiere den Inhalt des Informationsblatts, das ich zu der oben genannten Studie erhalten habe. Ich hatte genug Zeit, um meine Entscheidung zu treffen.
- Ich habe zufriedenstellende Antworten auf die Fragen erhalten, die ich im Zusammenhang mit meiner Teilnahme an der Studie gestellt habe. Ich bewahre das Informationsblatt auf und erhalte eine Kopie meiner schriftlichen Einverständniserklärung.
- Ich akzeptiere, dass mein Arzt über meine Teilnahme an der Studie informiert wird.
- Ich werde über Entdeckungen informiert, die sich direkt auf meine Gesundheit auswirken. Wenn ich diese Informationen nicht erhalten möchte, benachrichtige ich den Studienverantwortlichen.
- Ich kann jederzeit und ohne Angabe von Gründen meine Einwilligung zur Teilnahme an der Studie widerrufen, ohne dass dies die Fortsetzung meiner Betreuung beeinträchtigt.
- Mir wurde mitgeteilt, dass eine Versicherung abgeschlossen wurde, um die Schäden abzudecken, die ich aufgrund der Studie erleiden könnte.
- Mir ist bekannt, dass die im Informationsblatt für die Teilnehmer genannten Verpflichtungen während der gesamten Studiendauer eingehalten werden müssen. Die Leitung der Studie kann mich im Interesse meiner Gesundheit jederzeit ausschließen.

| Ort, Datum | Unterschrift des Teilnehmers |
| --- | --- |
| | |

# Bewertung der Auswirkung der Präsenz eines Aquariums im Wartebereich auf ältere Patienten und Empfangspersonal

Diese Studie wird von der Klinik für Allgemein-, Behinderten- und Seniorenzahnmedizin (ABS), am Zentrum für Zahnmedizin in Zürich (ZZM) der Universität Zürich (UZH), in Zürich, Schweiz, organisiert.

Geschätzte Mitarbeiterin,

Wir laden Sie ein, an unserem Forschungsprojekt teilzunehmen. Dieses Informationsblatt beschreibt unser Forschungsprojekt.

## 1. Ziele der Studie

Ziel dieser Studie ist es, die Auswirkung der Präsenz eines Aquariums im Wartebereich unserer Klinik auf ältere Patienten und Empfangspersonal zu bewerten. Zu diesem Zweck nehmen Sie an einer Umfrage mittels Fragebogen teil. Außerdem werden bei Ihnen Puls und Blutdruck gemessen, und Ihr Gemütszustand wird von einer Fachperson eingeschätzt.

## 2. Auswahl der Personen, die zur Teilnahme an der Studie berechtigt sind

Die Teilnahme steht allen Mitarbeitern offen, die an der Klinik für Alters-, Behinderten, und Seniorenzahnmedizin am Empfang tätig sind und gewillt sind an unserer Studie teil zunehmen.

## 3. Allgemeine Informationen zur Studie

- Diese Studie dient der Bewertung unseres neuen Aquariums im Wartebereich der ABS Klinik.
- Sie werden gebeten, uns einige Angaben zu Ihrer Person zu machen.
- Anschließend werden Ihnen mehrere Fragen zu Ihrem Gemütszustand gestellt.
- Wir führen diese Studie in Übereinstimmung mit den Anforderungen der Schweizer Gesetzgebung durch.
- Wir befolgen alle international anerkannten Richtlinien.

## 4. Ablauf für die Teilnehmer

Falls Sie sich zur Teilnahme entscheiden ist der Ablauf der Studie folgendermaßen strukturiert:

- Bevor Studienbeginn geben wir Ihnen Informationen zur Teilnahme an der Studie.
- Entschließen Sie sich zur Teilnahme an der Studie bitten wir Sie uns einige Angaben zu Ihrer Person zu machen.
- Anschließend geben Sie an, welches Ambiente Sie im Wartebereich unserer Klinik vorfinden.
- Wir bitten Sie dann, uns Ihren Gemütszustand, sowie Ihre allgemeine Erregtheit, mittels der zur Verfügung gestellten Fragebögen mitzuteilen.

## 5. Vorteile für die Teilnehmer

Die Teilnahme an der Studie ist kostenlos. Ihre Meinung ist uns wichtig.

## 6. Rechte der Teilnehmer

Sie nehmen nach eigenem Ermessen an dieser Studie teil. Wenn Sie sich dafür entscheiden, nicht teilzunehmen, oder wenn Sie sich dazu entschließen, Ihre Entscheidung im Verlauf der Studie zu überdenken, müssen Sie Ihre Ablehnung nicht begründen. Sie können jederzeit alle Fragen stellen, die für Ihr Verständnis der Studie erforderlich sind. Bitte wenden Sie sich an die Person, die am Ende dieses Informationsblatts angegeben ist.

**7. Pflichten der Teilnehmer**

Als Teilnehmer an der Studie erlauben Sie:

- Auskunft über Ihren aktuellen Gemütszustand, mittels Frageboden zu geben.

**8. Risiken und Einschränkungen für die Teilnehmer**

Die Studie ist nicht invasiv und birgt kein besonderes Risiko.

**9. Erkenntnisse während der Studie**

Der Prüfer wird Sie während der Studie über alle neuen Erkenntnisse informieren, die den Nutzen der Studie oder Ihre Sicherheit beeinträchtigen können, und daher Ihre Zustimmung zur Teilnahme. Sie werden mündlich und schriftlich informiert.

**10. Vertraulichkeit von Daten und Proben**

Zu Forschungszwecken gesammelte Daten werden während der Sammlung verschlüsselt. Codierung bedeutet, dass alle Daten, die Sie identifizieren (z. B. Name, Geburtsdatum usw.), durch einen Code ersetzt werden, sodass Personen, die diesen Code nicht kennen, diese Daten nicht mit einer anderen Person verknüpfen können. Der Code bleibt dauerhaft am Zentrum für Zahnmedizin. Alle an der Studie in irgendeiner Weise beteiligten Personen sind zur Wahrung des Berufsgeheimnisses verpflichtet. Wir garantieren die Einhaltung aller Datenschutzrichtlinien und zeigen Ihren Namen in keinem Print- oder Online-Bericht oder in keiner Veröffentlichung an. Sie haben jederzeit das Recht, Ihre Daten einzusehen.

**11. Rücktritt vom Studium**

Sie können jederzeit von der Studie zurücktreten, wenn Sie dies wünschen. Die bisher gesammelten Daten werden weiterhin analysiert, um den Wert der Studie insgesamt nicht zu beeinträchtigen.

**12. Entschädigung der Teilnehmer**

Ihre Teilnahme hat keine finanziellen Konsequenzen für Sie.

**13. Finanzierung der Studie**

Die Studie wird vollständig aus Mitteln finanziert, die der Klinik für Allgemein, Behinderten- und Seniorenzahnmedizin, Zentrum für Zahnmedizin, Zürich Schweiz, gewährt wurden.

**14. Kontakt (e)**

Im Zweifelsfall, bei Ängsten oder Notfällen während oder nach dem Studium können Sie sich jederzeit an eine der folgenden Personen wenden:

Untersuchungszahnärzte: med. dent. Andrea Lundberg, med. dent. Nicole Kalberer.

**15. Einverständniserklärung**

Schriftliche Einverständniserklärung zur Teilnahme an einem Forschungsprojekt.

Bitte lesen Sie dieses Formular sorgfältig durch. Sie können gerne Fragen stellen, wenn Sie diese nicht verstehen oder eine Erklärung wünschen.

**Titel der Studie:**
Bewertung der Auswirkung der Präsenz eines Aquariums im Wartebereich auf ältere Patienten und Empfangspersonal

**Verantwortliche Institution:**
Klinik für Allgemein-, Behinderten- und Seniorenzahnmedizin (ABS)
Zentrum für Zahnmedizin (ZZM)
Universität Zürich (UZH)
Plattenstrasse 11
CH-8032 Zürich | Schweiz

**Abteilung von**
Professor Murali SRINIVASAN
E-Mail: Murali.Srinivasan@zzm.uzh.ch

**Für das Projekt an Ort verantwortliche Zahnärzte:**
Med. dent. Andrea Lundberg, med. dent. Nicole Kalberer

**Teilnehmer / Teilnehmer:** ______________________________________________
(Vor- und Nachname in Druckbuchstaben)

**Geburtsdatum:**______________________________________________________

**O Frau  O Mann**

- Ich erkläre, von dem für diese Studie verantwortlichen Prüfer/Zahnarzt mündlich oder schriftlich über die Ziele und den Verlauf der Studie sowie über die Auswirkungen, Vorteile, möglichen Nachteile und potenziellen Risiken informiert worden zu sein.
- Ich nehme freiwillig an dieser Studie teil und akzeptiere den Inhalt des Informationsblatts, das ich zu der oben genannten Studie erhalten habe. Ich hatte genug Zeit, um meine Entscheidung zu treffen.
- Ich habe zufriedenstellende Antworten auf die Fragen erhalten, die ich im Zusammenhang mit meiner Teilnahme an der Studie gestellt habe. Ich bewahre das Informationsblatt auf und erhalte eine Kopie meiner schriftlichen Einverständniserklärung.
- Ich akzeptiere, dass mein Arzt über meine Teilnahme an der Studie informiert wird.
- Ich werde über Entdeckungen informiert, die sich direkt auf meine Gesundheit auswirken. Wenn ich diese Informationen nicht erhalten möchte, benachrichtige ich den Studienverantwortlichen.

- Ich kann jederzeit und ohne Angabe von Gründen meine Einwilligung zur Teilnahme an der Studie widerrufen, ohne dass dies die Fortsetzung meiner Betreuung beeinträchtigt.
- Mir wurde mitgeteilt, dass eine Versicherung abgeschlossen wurde, um die Schäden abzudecken, die ich aufgrund der Studie erleiden könnte.
- Mir ist bekannt, dass die im Informationsblatt für die Teilnehmer genannten Verpflichtungen während der gesamten Studiendauer eingehalten werden müssen. Die Leitung der Studie kann mich im Interesse meiner Gesundheit jederzeit ausschließen.

| Ort, Datum | Unterschrift des Teilnehmers |
| --- | --- |
| | |

**Zertifikat des Verantwortlichen:**

Hiermit bestätige ich, dass ich dem Teilnehmer Art, Bedeutung und Umfang der Studie erläutert habe. Ich erkläre, dass ich alle mit diesem Projekt verbundenen Verpflichtungen gemäß den geltenden Gesetzen erfülle. Sollte ich zu irgendeinem Zeitpunkt während des Abschlusses des Projekts Kenntnis von Elementen erlangen, die die Zustimmung des Teilnehmers zur Teilnahme am Projekt beeinträchtigen könnten, verpflichte ich mich, den Teilnehmer unverzüglich zu informieren.

| Ort, Datum | Name und Vorname des Prüfarztes (in Druckschrift)<br><br>Unterschrift des Verantwortlichen |
| --- | --- |
